# Identifying Differentially Expressed Genes of Zero Inflated Single Cell RNA Sequencing Data Using Mixed Model Score Tests

**DOI:** 10.3389/fgene.2021.616686

**Published:** 2021-02-05

**Authors:** Zhiqiang He, Yueyun Pan, Fang Shao, Hui Wang

**Affiliations:** ^1^Department of Biostatistics, School of Public Health, Nanjing Medical University, Nanjing, China; ^2^First Clinical Medical College, Nanjing Medical University, Nanjing, China; ^3^Department of Maternal and Child Health, School of Public Health, Peking University Health Science Center, Beijing, China

**Keywords:** score test, generalized linear mixed model, zero inflation, observational weights, differential expression analyses, single cell RNA sequencing

## Abstract

Single cell RNA sequencing (scRNA-seq) allows quantitative measurement and comparison of gene expression at the resolution of single cells. Ignoring the batch effects and zero inflation of scRNA-seq data, many proposed differentially expressed (DE) methods might generate bias. We propose a method, single cell mixed model score tests (scMMSTs), to efficiently identify DE genes of scRNA-seq data with batch effects using the generalized linear mixed model (GLMM). scMMSTs treat the batch effect as a random effect. For zero inflation, scMMSTs use a weighting strategy to calculate observational weights for counts independently under zero-inflated and zero-truncated distributions. Counts data with calculated weights were subsequently analyzed using weighted GLMMs. The theoretical null distributions of the score statistics were constructed by mixed Chi-square distributions. Intensive simulations and two real datasets were used to compare edgeR-zinbwave, DESeq2-zinbwave, and scMMSTs. Our study demonstrates that scMMSTs, as supplement to standard methods, are advantageous to define DE genes of zero-inflated scRNA-seq data with batch effects.

## Introduction

In modern biology, transcriptomics has been widely used to elucidate the molecular basis of biological processes and diseases (Van den Berge et al., 2018). Previous transcriptome sequencing techniques (bulk RNA-seq) (Wang et al., 2009) might obscure the cell type heterogeneity in different samples. Because of the resolution, bulk RNA-seq hardly defines the rare cells, such as stem cells and tumor cells. Single cell RNA sequencing (scRNA-seq) enables researchers to study characteristics of gene expression in the resolution of individual cells (Kolodziejczyk et al., 2015). scRNA-seq has been treated as an effective method to study cellular heterogeneity in complex biological systems, and is being applied by more researchers in various biological processes, such as stem cell development and differentiation, embryonic organ development, tumors, immunology, and neurology (Tang et al., 2009; McEvoy et al., 2011; Zeisel et al., 2015; Chu et al., 2016; Papalexi and Satija, 2018; Sun et al., 2019). Identifying differentially expressed (DE) genes is one of the most common analysis of both bulk RNA-seq and of scRNA-seq analysis (Robinson et al., 2010; Van den Berge et al., 2017, 2018; Sun et al., 2018).

For bulk RNA-seq and scRNA-seq data, batch effects conventionally were treated as the non-biological differences that occurs when samples or cells are measured in distinct batches. The measure of transcriptome can be influenced by different environments for cells (Luecken and Theis, 2019). Various methods to correct batch effects and preserve biological variability have been presented. Some methods directly remove or correct batch effects using linear models (Johnson et al., 2007; Tung et al., 2017; Somekh et al., 2019). ComBat (Johnson et al., 2007) is an empirical Bayes method which takes batch effects into a linear regression model of gene expression. ComBat was recommended for batch correction when groups or cell types and state compositions between batches are consistent (Luecken and Theis, 2019). Mutual nearest neighbors (MNNs) (Haghverdi et al., 2018) and canonical correlation analysis (CCA) (Butler et al., 2018) remove batch effects using nonlinear models. A method comparison study showed ComBat was the best one for both bulk RNA-seq and scRNA-seq data (Büttner et al., 2019). For DE analysis, it was recommended that DE testing should be conducted on measure data with covariates including the batch information in the model design, not on batch corrected data (Luecken and Theis, 2019).

Some studies directly used traditional bulk RNA-seq DE methods (Krieg et al., 2018; Roerink et al., 2018; Li et al., 2019; Mehtonen et al., 2020). Limma-voom (Ritchie et al., 2015) applies weighted linear regression models for log-transformed count data. edgeR (Robinson et al., 2010; McCarthy et al., 2012) and DESeq2 (Love et al., 2014) model the gene expression count data based on generalized linear models (GLMs) under negative binomial (NB) distributions. It was demonstrated that NB models overestimated the dispersion parameter with excess zero counts, which influenced the power to DE analysis (Van den Berge et al., 2018). Different to bulk RNA-seq data, dropout events cause excess zeros for scRNA-seq read count data (Finak et al., 2015; Hashimshony et al., 2016). Therefore, zero inflation or an excess of zeros is a particular feature of scRNA-seq data, and it is not considered for these methods. SCDE (Kharchenko and Fan, 2019) and MAST (Finak et al., 2015; McDavid et al., 2019) model the redundant zeros of scRNA-seq data by zero inflation and hurdle models, respectively. Both zinbwave (Risso et al., 2018; Van den Berge et al., 2018) and zingeR (Van den Berge et al., 2017) estimates observational weights based on a zero-inflated negative binomial (ZiNB) model and downweight excess zeros followed by classical bulk RNA-seq DE tools (e.g., edgeR and DESeq2). The performance of two combinations, edgeR-zinbwave and DESeq2-zinbwave, outperform other DE methods (Van den Berge et al., 2018).

Here, based on isoVCT (Yang et al., 2017) and SMMATs (Chen et al., 2019), we implement a series of efficient methods, the single cell mixed model score tests (scMMSTs), to identify DE genes for defined cell types in scRNA-seq data considering batch effects and zero inflation. isoVCT, a DE method for bulk RNA-seq, uses a random effect to consider the heterogeneous isoform effects. In large-scale whole-genome sequencing (WGS) studies, SMMATs are powerful and computationally efficient variant set tests for continuous and binary traits, which integrates the burden test and SKAT (Wu et al., 2011) under the framework of generalized linear mixed models (GLMMs).

## Methods

### Generalized Linear Mixed Models

For a single gene, we consider the following:

$$g\,\left(\mu_{i}\right)=\alpha +g_{i}\,{\text{B}}_{i}\,\beta +{\text{B}}_{i}\,\text{b},$$

where *g*(⋅) is a monotonic differentiable link function for GLMs, μ_*i*_ = *E*(*y*_*i*_|*g*_*i*_,**B**_*i*_,**b**) denotes the mean of phenotype or count *y_i* for subject or cell *i* for a given gene with sample size *n* to the intercept α, *g_i* is the group, cluster or cell type covariate dummy variable binary value for subject *i*, **B**_*i*_is the row vector of dummy variables values of the batch or individual covariate for subject *i*, β is the group effects associated with bathes and **b** is the batch effects. In the above equation, the group effects β are assumed to follow the normal distribution$N\,\left(\beta_{0}\,1_{p},\sigma_{\beta }^{2}\,{\text{I}}_{p}\right)$, where **1**_*p*_ is the *p*×1 dimensional vector whose elements are all 1, **I**_*p*_ is the *p*×*p* dimensional identity matrix, β_0_ and $\sigma_{\beta }^{2}$ are mean and variance of the normal distribution and *p* is the number of batches. If $\sigma_{\beta }^{2}>0$, group effects are associated with the batches. We assume the batch random effects $\text{b}∼N\,\left(0_{p},\sigma_{b}^{2}\,{\text{I}}_{p}\right)$, where **0**_*p*_ is the *p*×1 dimensional vector whose elements are all 0 and $\sigma_{b}^{2}$ is the variance. We consider the binomial, quasi-binomial, Poisson, quasi-Poisson, and NB distributions to model*yi*. Binary phenotypes are commonly modeled by binomial and quasi-binomial distributions and counts are commonly modeled by Poisson, quasi-Poisson, and NB distributions.

For single cell RNA-seq data of a given gene, *y_i* is the count for cell *i*. We identify DE genes for each defined cell type in the form of one-against-others, so*gi*, the cell type covariate for cell*i*, is binary. GLMMs under Poisson, quasi-Poisson and NB distributions are appropriate in this scenario.

### Single Cell Mixed Model Score Tests

Testing *H*_0_:β = **0** is equivalent to testing *H*_0_:β_0_ = 0 and$\sigma_{\beta }^{2}=0$. Under the null hypothesis, the reduced GLMM is as follows.

$$g\,\left(\mu_{0\,i}\right)=\alpha +{\text{B}}_{i}\,\text{b},$$

where μ_0*i*_ = *E*(*y*_*i*_|μ_0_,*b*_*i*_).

We construct a variance component score test statistic *T* derived by testing $H_{0}^{\prime }:\sigma_{\beta }^{2}=0$ under the assumptionβ0 = 0. SMMAT-O was also derived in the same manner. Under $H_{0}^{\prime }$ with the assumptionβ0 = 0, we have the same reduced null model as that under*H*0:β = **0**. Therefore, our derived test statistic *T* is applicable for testing*H*0. The test statistic *T* is shown as follows.

$$T=\frac{{\left(\text{y}-{\hat{\mu }}_{0}\right)}^{T}\,\hat{\Phi }\,{\text{G}}_{B}\,{\text{G}}_{B}^{T}\,\hat{\Phi }\,\left(\text{y}-{\hat{\mu }}_{0}\right)}{\hat{\tau }},$$

where **y** = (*y*_1_*y*_2_⋯*y*_*n*_)*T* is an *n*×1 vector of counts or phenotypes, ${\hat{\mu }}_{0}=g^{-1}\,\left(\hat{\alpha }+{\text{B}}_{i}\,\hat{\text{b}}\right)$ is the estimated mean vector of the reduced null model under *H_0*,$\hat{\alpha }$ and $\hat{\text{b}}$ are estimates of the α and **b**, $\Phi =d\,i\,a\,g\,\left\{1/\left(1+\left({\hat{\mu }}_{0\,i}/\hat{\theta }\right)\right)\right\}$ for the NB distribution with the estimated dispersion parameter $\hat{\theta }$ and $\hat{\Phi }={\text{I}}_{n}$ for other distributions mentioned, $\text{B}={\left({\text{B}}_{1}^{T}\,{\text{B}}_{2}^{T}\,\cdots \,{\text{B}}_{n}^{T}\right)}^{T}$ is an *n*×*p* design matrix of group covariate dummy variables values, ${\text{G}}_{B}={\left(g_{1}\,{\text{B}}_{1}^{T}\,g_{2}\,{\text{B}}_{2}^{T}\,\cdots \,g_{n}\,{\text{B}}_{n}^{T}\right)}^{T}$ is an *n*×*p* design matrix of interactions of group and batch covariates with the multiplication of corresponding dummy variables values and $\hat{\tau }$ is the estimate of dispersion parameter τ for quasi distributions, which is 1 for the binomial, Poisson and NB distributions and is estimated by the residual deviance divided by the degree of freedom of the reduced null model for quasi-binomial and quasi-Poisson distributions.

The asymptotic distribution of the statistic *T* under *H_0* is derived as follows. Following the theoretical results of mixed models (Harville, 1977; Breslow and Clayton, 1993; Santos Nobre and da Motta Singer, 2007; Chen et al., 2016), we have $\hat{\text{e}}=\left(\text{y}-{\hat{\mu }}_{0}\right)/\sqrt{\hat{\tau }}$ asymptotically following a n-dimensional multivariate normal distribution $\text{M}\,V\,N_{n}\,\left(0,{\hat{\text{D}}}^{-1}\,\hat{\text{V}}\,\hat{\text{P}}\,\hat{\Sigma }\,\hat{\text{P}}\,\hat{\text{V}}\,{\hat{\text{D}}}^{-1}\right)$ under *H*_0_, where $\hat{\text{D}}=d\,i\,a\,g\,\left\{g^{\prime }\,\left({\hat{\mu }}_{0\,i}\right)\right\}$, whose diagonal elements are the first order derivative of the link function *g*(⋅) evaluated at ${\hat{\mu }}_{0\,i}$, $\hat{\text{P}}$ is the *n*×*n* projection matrix of the reduced null model $\hat{\text{P}}={\hat{\Sigma }}^{-1}-{\hat{\Sigma }}^{-1}\,1_{n}\,{\left(1_{n}^{T}\,{\hat{\Sigma }}^{-1}\,1_{n}\right)}^{-1}\,1_{n}^{T}\,{\hat{\Sigma }}^{-1}$ with $\hat{\Sigma }=\hat{\text{V}}+{\hat{\sigma }}_{b}^{2}\,{\text{BB}}^{T}$, $\hat{\text{V}}=d\,i\,a\,g\,\left\{{\left(g^{\prime }\,\left({\hat{\mu }}_{0\,i}\right)\right)}^{2}\,\hat{V\,a\,r}\,\left(y_{i}\right)\right\}$, the first order derivative function of the link function *g*′(⋅) and the estimated variance of *y_i*, $\hat{V\,a\,r}\,\left(y_{i}\right)$. For binomial and quasi-binomial distributions, ${\left(g^{\prime }\,\left({\hat{\mu }}_{0\,i}\right)\right)}^{2}\,\hat{V\,a\,r}\,\left(y_{i}\right)=1/\left[{\hat{\mu }}_{0\,i}\,\left(1-{\hat{\mu }}_{0\,i}\right)\right]$. For Poisson and quasi-Poisson distributions, ${\left(g^{\prime }\,\left({\hat{\mu }}_{0\,i}\right)\right)}^{2}\,\hat{V\,a\,r}\,\left(y_{i}\right)=1/{\hat{\mu }}_{0\,i}$. For NB distributions, ${\left(g^{\prime }\,\left({\hat{\mu }}_{0\,i}\right)\right)}^{2}\,\hat{V\,a\,r}\,\left(y_{i}\right)=\left(1/{\hat{\mu }}_{0\,i}\right)+\left(1/\hat{\theta }\right)$. Since $\hat{\text{P}}\,\hat{\Sigma }\,\hat{\text{P}}=\hat{\text{P}}$ and $\hat{\Phi }={\hat{\text{V}}}^{-1}\,\hat{\text{D}}$, the asymptotic distribution can be simplified as **M***VN*_**n**_$\left(0,{\hat{\Phi }}^{-1}\,\hat{\text{P}}\,{\hat{\Phi }}^{-1}\right)$. Therefore, under*H*0, *T*, a quadratic form of $\hat{\text{e}}$, asymptotically follows a mixture Chi-square distribution $\sum_{i=1}^{p}\xi_{i}\,\chi_{1,i}^{2}$, where $\chi_{1,i}^{2}$ are independent Chi-square distributions with 1 degree of freedom, and *ξ*_*i*_ are the eigenvalues of $\text{E}={\text{G}}_{B}^{T}\,\hat{\text{P}}\,{\text{G}}_{B}$. Notably, $\hat{\Sigma }$ in $\hat{\text{P}}$ has a simple structure which makes ${\hat{\Sigma }}^{-1}$ to be solved explicitly and E to be calculated efficiently. The *p*-value of the test can be calculated soon after the estimation of the reduced null model. More details of the computational efficiency of scMMSTs are discussed in section “Performance Evaluation”. The estimation procedure of ${\hat{\mu }}_{0\,i}$ is the same for binomial and quasi-binomial distribution pair and the Poisson and quasi-Poisson distribution pair. Thus, we implement quasi distributions to allow flexibility. In the followings, unless specified otherwise, “binomial” stands for both binomial and quasi-binomial and “Poisson” stands for both Poisson and quasi-Poisson.

There is zero inflation in scRNA-seq count data. Therefore, following the idea of ZINB-WaVE, a weighting strategy is implemented. Firstly, observational weights are calculated for all counts independently with details shown in sections “Zero-Inflated and Zero-Truncated Distributions for Counts” and “Calculations of Observational Weights for scMMSTs.” Afterward, counts data with calculated weights are analyzed under the weighted GLMMs. Accordingly, a weighted version test statistic *T_w* for scMMSTs is proposed as follows with above notations.

$$T_{w}=\frac{{\left(\text{y}-{\hat{\mu }}_{0}\right)}^{T}\,\hat{\Phi }\,{\text{WG}}_{B}\,{\text{G}}_{B}^{T}\,\text{W}\,\hat{\Phi }\,\left(\text{y}-{\hat{\mu }}_{0}\right)}{\hat{\tau }},$$

where **W** = *diag*{*w*_*i*_}and *w_i* is the given weights for count *y_i*. The estimation is based on the weighted GLLMs for the reduced null model. We denote $1_{w,n}={\text{W}}^{\frac{1}{2}}\,1_{n}$, ${\text{B}}_{w}={\text{W}}^{\frac{1}{2}}\,\text{B}$, ${\hat{\text{V}}}_{w}={\text{W}}^{-\frac{1}{2}}\,\hat{\text{V}}\,{\text{W}}^{-\frac{1}{2}}$, ${\hat{\Sigma }}_{w}=\hat{\text{V}}+{\hat{\sigma }}_{b}^{2}\,{\text{B}}_{w}\,{\text{B}}_{w}^{T}$, ${\tilde{\hat{\Sigma }}}_{w}={\text{W}}^{-\frac{1}{2}}\,{\hat{\Sigma }}_{w}\,{\text{W}}^{-\frac{1}{2}}$ and ${\hat{\text{P}}}_{w}={\text{W}}^{\frac{1}{2}}\,{\hat{\Sigma }}_{w}^{-1}\,{\text{W}}^{\frac{1}{2}}-{\text{W}}^{\frac{1}{2}}\,{\hat{\Sigma }}_{w}^{-1}\,1_{w,n}\,{\left(1_{w,n}^{T}\,{\hat{\Sigma }}_{w}^{-1}\,1_{w,n}\right)}^{-1}\,1_{w,n}^{T}\,{\hat{\Sigma }}_{w}^{-1}\,{\text{W}}^{\frac{1}{2}}={\tilde{\hat{\Sigma }}}_{w}^{-1}-{\tilde{\hat{\Sigma }}}_{w}^{-1}\,1_{n}\,{\left(1_{n}^{T}\,{\tilde{\hat{\Sigma }}}_{w}^{-1}\,1_{n}\right)}^{-1}\,1_{n}^{T}\,{\tilde{\hat{\Sigma }}}_{w}^{-1}$. Based on the theoretical results of weighted GLMMs (Harville, 1977; Breslow and Clayton, 1993; Santos Nobre and da Motta Singer, 2007; Chen et al., 2016), if *H_0* and **W** are true, we have $\hat{\text{e}}$ asymptotically normally distributed as $\text{M}VN_{n}(0,{\hat{\text{D}}}^{-1}{\hat{\text{V}}}_{w}{\hat{\text{P}}}_{w}$${\tilde{\hat{\Sigma }}}_{w}{\hat{\text{P}}}_{w}{\hat{\text{V}}}_{w}{\hat{\text{D}}}^{-1})$. Since ${\hat{\text{P}}}_{w}\,{\tilde{\hat{\Sigma }}}_{w}\,{\hat{\text{P}}}_{w}={\hat{\text{P}}}_{w}$, $\hat{\Phi }={\hat{\text{V}}}^{-1}\,\hat{\text{D}}$ and ${\hat{\text{D}}}^{-1}\,{\hat{\text{V}}}_{w}={\hat{\text{D}}}^{-1}\,{\text{W}}^{-\frac{1}{2}}\,\hat{\text{V}}\,{\text{W}}^{-\frac{1}{2}}={\hat{\Phi }}^{-1}\,{\text{W}}^{-1}$, where ${\text{W}}^{-\frac{1}{2}},{\hat{\text{D}}}^{-1},\hat{\text{V}}$ are diagonal matrices, the asymptotic distribution can be simplified as $\text{M}VN_{n}(0,{\hat{\Phi }}^{-1}{\text{W}}^{-1}$${\hat{\text{P}}}_{w}{\text{W}}^{-1}{\hat{\Phi }}^{-1})$. If *H_0* and **W** are true, *T*_*w*_, a quadratic form of $\hat{\text{e}}$, asymptotically follows a mixture Chi-square distribution $\sum_{i=1}^{p}\xi_{i}\,\chi_{1,i}^{2}$, where $\chi_{1,i}^{2}$ are independent Chi-square distributions with 1 degree of freedom, and *ξ*_*i*_ are the eigenvalues of ${\text{E}}_{w}={\text{G}}_{B}^{T}\,{\hat{\text{P}}}_{w}\,{\text{G}}_{B}$. Note that ${\hat{\Sigma }}_{w}$ in ${\hat{\text{P}}}_{w}$ does not have the simple structure of $\hat{\Sigma }$, which makes it hard to analytically and explicitly solve ${\hat{\Sigma }}_{w}^{-1}$. Therefore, we propose ${\text{E}}_{w}^{\prime }={\text{G}}_{B}^{T}\,\text{W}\,\hat{\text{P}}\,{\text{WG}}_{B}$ to approximate E**_w_** for simplicity and efficiency, where we treat $\hat{\text{e}}$ as it is estimated by GLMMs without weights. Calculated weights are 1 for nonzero counts and between 0 and 1 for zero counts. Thus, this approximation performs worse when there are more redundant zeros, which might influence the performance of scMMSTs.

### Zero-Inflated and Zero-Truncated Distributions for Counts

#### Zero-Inflated Distributions for Counts

A zero-inflated distribution for counts is a mixture distribution with two components, which are a point mass at zero and a conventional random variable distribution for counts, e.g., Poisson and NB distributions. The probability mass function (pmf) of a zero-inflated distribution for counts is as follows.

$$f_{Z\,I}\,\left(y;\theta ,\pi \right)=\pi \,\delta_{0}\,\left(y\right)+\left(1-\pi \right)\,f\,\left(y;\theta \right),\forall y\in \mathbb{N},$$

where π ∈ [0,1] indicates the probability of zero inflation, δ_0_(⋅) the Dirac function, *f*(⋅;θ) the pmf of a conventional distribution with parameter vector θ. The observational weights of the counts can be calculated under a zero-inflated distribution model as the conditional probability that a given count *y* belongs to the conventional distribution with parameter estimates $\hat{\theta },\hat{\pi }$:

$$w=\frac{\left(1-\hat{\pi }\right)\,f\,\left(y;\hat{\theta }\right)}{f_{Z\,I}\,\left(y;\hat{\theta },\hat{\pi }\right)}.$$

Note that *w* is 1 for nonzero counts and ∈ (0,1) for zeros counts. All the weights for counts under the conventional distribution are 1. Under a zero-inflated distribution, we take the weights of nonzero counts remain 1 and downweight zero counts from 1 to the conditional probability that a given count *y* belongs to the conventional distribution. Counts with observational weights are subsequently analyzed under the weighted version of models for the conventional distribution. In ZINB-WaVE, this weighting strategy is applied and the above formula is applied to calculate observational weights under the ZiNB distribution (Van den Berge et al., 2018).

#### Zero-Truncated Distributions for Counts

A zero-truncated distribution for counts is a distribution for counts with random variable values truncated at zero, i.e., only counts larger than zero can be observed. In the followings, we refer to zero-truncated distributions as truncated distributions for short. The pmf of a truncated distribution for counts is as follows.

$$f_{T\,r}\,\left(y;\theta \right)=\frac{f\,\left(y;\theta \right)}{P_{f}\left(t>0;\theta \right)}=\frac{f\,\left(y;\theta \right)}{\sum_{t=1}^{+\infty }f\,\left(t;\theta \right)},\forall y\in {\mathbb{N}}_{+},$$

where *f*(⋅;θ) denotes the pmf of a conventional distribution for counts with parameter vectorθ. The observational weights of nonzero counts are 1 and weights of zero counts can be calculated under a truncated distribution model as following:

$$w=\frac{n_{1}f\left(y=0;\hat{\theta }\right)}{n_{0}\,\sum_{t=1}^{+\infty }f\,\left(t;\hat{\theta }\right)},$$

where *n_1* is the number of nonzero counts, *n_0* is the number of the zero counts in the whole sample and $\hat{\theta }$ is the parameter vector estimate.

The derivation of the above formula is as follows. Nonzero counts follow the truncated distribution with parameter θ which is the also the parameter for the corresponding conventional distribution. Therefore, the probability of zero counts is estimated as $f\left(y=0;\hat{\theta }\right)$. All the weights for counts under the conventional distribution are 1. However, since excess zeros are presented, the observational weights of nonzero counts remain 1 and zero counts are reweighted from 1 to *w*, so that $\frac{w\cdot n_{0}}{w\cdot n_{0}+1\cdot n_{1}}=f\left(y=0;\hat{\theta }\right)$. The resulting formula for observational weights *w* is derived by solving the equation. Counts are then analyzed with observational weights calculated under the weighted version of models for the conventional distribution.

### Calculations of Observational Weights for scMMSTs

In ZINB-WaVE, the weighting strategy shown in the previous section is applied and observational weights are estimated by the ZiNB regression (Van den Berge et al., 2018). For our methods, the truncated Poisson (TrPois), zero-inflated Poisson (ZiPois), truncated negative binomial (TrNB), and ZiNB distributions are considered. Following the weighting strategy mentioned and *H*_0_:β = **0**, we estimate parameters for counts in each batch and calculate the weights accordingly using the formulas in section “Zero-Inflated and Zero-Truncated Distributions for Counts” for simplicity with the assumption of no group effects.

For zero-inflated distributions, weights are the conditional probabilities that a count *y* belongs to the corresponding conventional distribution. We directly use ZINB-WaVE for the ZiNB distribution, and implement the algorithm in Appendix A of the paper (Böhning et al., 1999) for the ZiPois distribution. In ZINB-WaVE, no mixed models are involved. Thus, we treat batch effects as fixed effects in the ZiNB regression without group effects to calculate weights using all counts data, when using ZINB-WaVE. For TrPois distribution, since the pmf $f_{T\,r\,P\,o\,i\,s}\,\left(y\right)=\frac{f_{P\,o\,i\,s}\,\left(y\right)}{1-e^{-\lambda }}=\frac{\lambda^{y}\,e^{-\lambda }}{y!\,1-e^{-\lambda }}$, we can derive the method of moment estimate and maximum likelihood estimate $\hat{\lambda }$ and they are identical by numerically solve the equation $\frac{\hat{\lambda }}{1-e^{-\hat{\lambda }}}=\bar{y}$, where $\bar{y}$ is the sample mean for the truncated sample. For each batch, the weights are $w_{i}=\frac{n_{1}\,e^{-\hat{\lambda }}}{n_{0}\,\left(1-e^{-\hat{\lambda }}\right)}$ for a zero count and *w_i=1* for nonzero *y_i*, where *n_1* is truncated sample size for the batch and *n_0* is the number of the zero counts in the batch. TrPois and ZiPois perform very close to each other. For TrNB distribution, we implement the formulas in section “Results” of the paper (Rider, 1955) to estimate the mean parameter μ and the dispersion parameterθ for each batch. The common dispersion parameter θ is estimated by the harmonic mean of the estimated $\hat{\theta }$ for each batch. However, this algorithm is not robust for small θ (θ < 2, based on simulations). The weights are $w_{i}=\frac{n_{1}\,{\left(\hat{\theta }/\left(\hat{\theta }+\hat{\mu }\right)\right)}^{\hat{\theta }}}{n_{0}\,\left(1-{\left(\hat{\theta }/\left(\hat{\theta }+\hat{\mu }\right)\right)}^{\hat{\theta }}\right)}$ for zero counts in each batch, where $\hat{\theta }$ and $\hat{\mu }$ are respectively the estimated dispersion and mean parameters for the NB distribution using counts in the batch, and *w_i=1* for nonzero *y_i* for each corresponding batch.

After weights are calculated, counts data with weights are analyzed under weighted GLMMs shown in section “Single Cell Mixed Model Score Tests.” Note that weights are calculated independently of GLMMs. Theoretically, the weights are 1 under conventional distributions. The calculated observational weights for nonzero counts remain 1. If there are calculated weights of zero counts far from 1 and closer to 0, it indicates that there are excess zeros. If calculated weights of zero counts are close to 1, the results for conventional distributions are similar to those considering zero inflation. In ZiNB-Wave, weights are calculated through the ZiNB regressions on all counts. However, the weights for TrPois, ZiPois, and TrNB are calculated using counts for each batch with smaller sample sizes. Therefore, although the calculation of weights for TrPois, ZiPois and TrNB is easier to implement and time saving, it is less accurate and less reliable than that for ZiNB-Wave and the performances of scMMSTs are affected.

### Performance Evaluation

Performances of DE methods considered are assessed in terms of the per-comparison error rate (PCER), which refers to type I error rate (i.e., the proportion of false positives), line plots of the true positive rate (TPR) vs. the false discovery proportion (FDP) and the areas under the receiver operating characteristic (ROC) curves [i.e., the TPR vs. the false positive rate (FPR) curves] (AUCs) with definitions as follows.

$$T\,P\,R=\frac{T\,P}{P},F\,P\,R=\frac{F\,P}{N},F\,D\,P=\frac{F\,P}{\text{max}\,{\left(1,F\,P+T\,P\right)}^{\prime }}$$

where we use the following abbreviations for empirical quantities: FP (the number of false positives), TP (the number of true positives), N (the number of negative samples), P (the number of positive samples). FDP-TPR curves for adjusted *p*-values are plotted by *iCOBRA* Bioconductor R package (version 1.12.1) (Soneson and Robinson, 2016) and AUCs for adjusted *p*-values are calculated by *pROC* R package (version 1.16.2) (Robin et al., 2011). Unless otherwise stated, the adjusted *p*-values for all DE methods considered are calculated by the Benjamini and Hochberg method (Benjamini and Hochberg, 1995) for FDR control.

### Comparison Methods

The 12 methods considered for comparisons are Poisson, TrPois, ZiPois, NB, TrNB, NB-zinb, DESeq2, DESeq2-zinb, edgeR, edgeR-zinb, limma-voom, and MAST. The first six methods are our implemented methods of scMMSTs s under GLMMs assumptions and the last six methods are the state-of-the-art DE methods, where Tr, Zi, Pois, NB, and zinb are abbreviations of truncated, zero-inflated, Poisson, ZINB-WaVE, respectively. We follow the implementations of the last six DE methods above in the *zinbwave* paper (Van den Berge et al., 2018) and the R packages used are *edgeR* (version 3.28.1), *DESeq2* (version 1.26.0), *limma* (version 3.42.2), *MAST* (version 1.12.0), and *zinbwave* (version 1.8.0), which was developed to deal with zero inflation for scRNA-seq data by a weighting strategy and was used in edgeR-zinb, DESeq2-zinb, and NB-zinb. The binomial distribution scMMST is implemented, however, not covered in the simulations and real data analysis since only methods for count data are considered in thisarticle.

The implementations of scMMSTs are available in Supplementary Data S1. Codes for simulations and real data analysis are partially based on the GitHub repositories^1^^2^ of papers (Yang et al., 2017; Van den Berge et al., 2018) and the *GMMAT* R package (version 1.3.0) (Chen et al., 2016, 2019). R packages *doParallel* (version 1.0.15) (Corporation and Weston, 2019) and *BiocParallel* (version 1.20.1) (Morgan et al., 2019) are used for parallel computation. The reduced null model is estimated by *lme4* R package (version 1.1.23) and *p*-values are calculated by *CompQuadForm* R package (version 1.4.3). Simulated single cell datasets are generated by *splatter* R package (version 1.10.1) (Zappia et al., 2017). Additionally, the code to reproduce all analyses, figures and tables reported in this manuscript is attached in Supplementary Data S1.

### Simulations

We perform simulations to evaluate performances of scMMSTs, which are our methods of association tests under the proposed GLMMs, comparing with state-of-art DE methods under a range of scenarios. We simulate the scRNA-seq data based on GLMMs directly and by the R package *splatter*. *Splatter* can directly estimate model parameters for real scRNA-seq data and generate quality controlled simulated mock datasets with DE genes easily and can add batch effects, which are not associated with group effects, to the simulated data. The simulated number of genes for one dataset by *splatter* and GLMMs is 10,000 and the number of cells is 250 with balanced two groups and five batches. In the DE genes simulations, the proportion of the DE genes is set to be 0.1.

Additional parameters of *splatter* simulations, batch.facLoc–batch factor location, batch.facScale–batch factor scale, and out.prob–the expression outlier probability, are set to be 0.5. For DE gene simulations, de.facLoc, DE factor location, is set to 2 and de.facScale, DE factor scale, is set to be 0.5.

The procedure to simulate datasets based on the proposed GLMMs is as follows. We assume that the scRNA-seq count data follow Poisson and NB distributions and generate *y_i* based on the GLMM shown with the parameters setting and generate a Bernoulli random variable *z_i* with parameterπ*i* = *logit*^−1^(μ_π_ + **B**_*i*_**b**). Larger values of parameter μ_π_ causes smaller baseline proportions of zeros. If *z*_*i*_ = 0, then *y*_*i*_ = 0, and *y_i* remains the same otherwise. The parameter settings for simulations are based on the real data analysis and references (Yang et al., 2017). Seven parameters are considered: the variance of the batch or individual effects $\text{b}\,\left(\sigma_{b}^{2}\right)$, the variance of the group or cell type effects $\beta \,\left(\sigma_{\beta }^{2}\right)$, the baseline group effect (β_0_), the number of batches (*p*), the dispersion parameter (θ = 1/ϕ) for NB distributions and the intercepts (μ_0_) and (μ_π_) for the GLMM and logstic regression for excess zeros, respectively. $\sigma_{b}^{2}$shows the heterogeneity of batch effects in different batches.$\sigma_{\beta }^{2}$shows the heterogeneity of group effects in different batches. β_0_ shows the baseline group effect. The larger the |β_0_|, the larger the baseline group effect is. Other parameters describe the features of the gene expression and zero inflation. $\sigma_{b}^{2}$ is set to be 0.25 and $\sigma_{\beta }^{2}$ varies in 0, 0.01, 0.25, and 1. β_0_ varies in 0, 0.01, 0.1, 0.3, and 0.5. θ varies in 0.5, 1, and 2. μ_π_ varies in −1, 0, and 2. *p=5* and μ0 = 5.

### Real Data Sets

#### Usoskin Dataset

This scRNA-seq dataset contains mouse neuronal cells in the dorsal root ganglion (Usoskin et al., 2015). The processed expression values were downloaded from the Github respiratory^3^ of the *zinbwave* paper. Following the process procedures given in the *zinbwave* paper, the authors considered 622 cells with a classification of 11 neuronal cell-types, which were denoted as NF1 to NF5, NP1 to NP3, PEP1, PEP2 and TH. Genes with less than 20 counts were removed and a total of 12,132 genes are considered for the following analyses with 68% zero counts. The authors showed the existence of a batch effect related to the picking session for the cells. Thus, the picking session covariate (with values Cold, RT-1, and RT-2) in this dataset was considered as a batch covariate for real data analysis. The batch effect was associated with expression measures and the relationship between zero inflation and sequencing depth, which was shown in Figure 5 of the *zinbwave* paper (Hicks et al., 2015; Van den Berge et al., 2018). We repeated the results of Figures 5A,B of the *zinbwave* paper in Supplementary Figures S1A,B. There is a large variation in the depth of sequencing among batches, which weaken the overall association with zero inflation when pooling cells across batches (Supplementary Figure S1A). Zero inflation was also identified for the Usoskin dataset. Histograms of observational weights for nonzero counts, which were calculated by the ZINB-WaVE model including the cell type as a covariate with and without the batch effect as fixed effects, are shown in Supplementary Figure S1B. Calculated weights of nonzero counts with and without the batch effect both have high modes near zero. This suggests zero inflation in the Usoskin dataset. The real data analysis of the processed Usoskin dataset was done to identify DE genes for defined 11 cell types vs. the rest. Simulated datasets based on this dataset were generated by *spaltter* with estimated corresponding parameters. For a null dataset without DE genes, we created 10,000 genes, 250 cells, five balanced batches and two balanced groups for cells. Twelve methods were implemented to identify DE genes between the two groups for each of the 30 simulated null data sets. A gene was declared to be DE if its unadjusted *p*-value was less than or equal to 0.05. Declared DE genes were false positives for these simulated null datasets. The empirical PCER of each method was calculated as the proportion of declared DE genes and was compared to the 0.05 nominal PCER.

#### Tung Dataset

This scRNA-seq dataset is for induced pluripotent stem cells from three individuals from HapMap (Tung et al., 2017). Following the *splatter* paper (Zappia et al., 2017), the matrix of molecules (UMIs) was treated as counts and was used directly. This dataset is available from GEO (accession GSE77288)^4^ and the Github respiratory^5^ of the *splatter* paper. No batch information is available for this dataset. Genes with less than 20 counts were removed and a total of 14,893 genes with 864 cells containing 44% zero counts were considered. Zero inflation was identified for the Tung dataset. Histograms of observational weights of nonzero counts of two filtered datasets (18,726 genes with more than 0 count and 14,893 genes with more than 19 counts, respectively), which were calculated by the ZINB-WaVE model, are shown in Supplementary Figures S1C,D. There are moderate proportion s of calculated weights of nonzero counts close to zero. This suggests zero inflation in the Tung dataset. Comparing to the Usoskin dataset, the Tung dataset is less zero inflated. We generated 30 simulated null datasets and identified DE genes using the same procedures for the Usoskin dataset with *spaltter*.

## Results

### Method Overview

Single cell mixed model score tests are computationally efficient DE analysis tools for scRNA-seq data considering batch effects and zero inflation. Bath effects are estimated as random effects under the reduced null models of GLMMs. A weighting strategy is implemented to characterize excess zeros. The score statistics are derived on theoretical asymptotic distributions. First, we estimated normalization factors of count matrix by the function *calcNormFactors* in *edgeR* after counts per million (CPM) normalization. Second, the estimation of the observational weights is efficient. We use *zinbwave* to fit NB-zinb which might be the most time-consumed assumption. Third, we use *lme4* for the estimation, the most efficient method to fit GLMM, to estimation the parameters in the null hypothesis (Eddelbuettel and François, 2011; Eddelbuettel, 2013; Eddelbuettel and Balamuta, 2017). Considering the real data, the estimation procedure of mixed model is not related to the number of groups or cell types. Compared to the traditional estimation procedure, scMMSTs use three strategies to decrease memory usage and computation time. First, scMMSTs do not need to store *n*×*n* matrices $\hat{\text{P}}$ and $\hat{\Sigma }$ explicitly. The *p*-value is efficiently calculated by *CompQuadForm* with eigenvalues of E or ${\text{E}}_{w}^{\prime }$, which is only a *p*×*p* matrix. Second, scMMSTs use an analytical form to calculate the inverse of $\hat{\Sigma }$ which might be the most time consumption procedure in the estimation of *T* or*Tw*. Third, scMMSTs is implemented for parallel computing. Therefore, although more complicated models GLMMs are considered, scMMSTs are computationally affordable compared to other DE methods.

### Simulations by Real Datasets and *Splatter*

Simulated datasets generated by the *splatter* used parameters estimated from two publicly available real scRNA-seq datasets, the Usoskin (Usoskin et al., 2015) and Tung (Tung et al., 2017) datasets.

**FIGURE 1:**
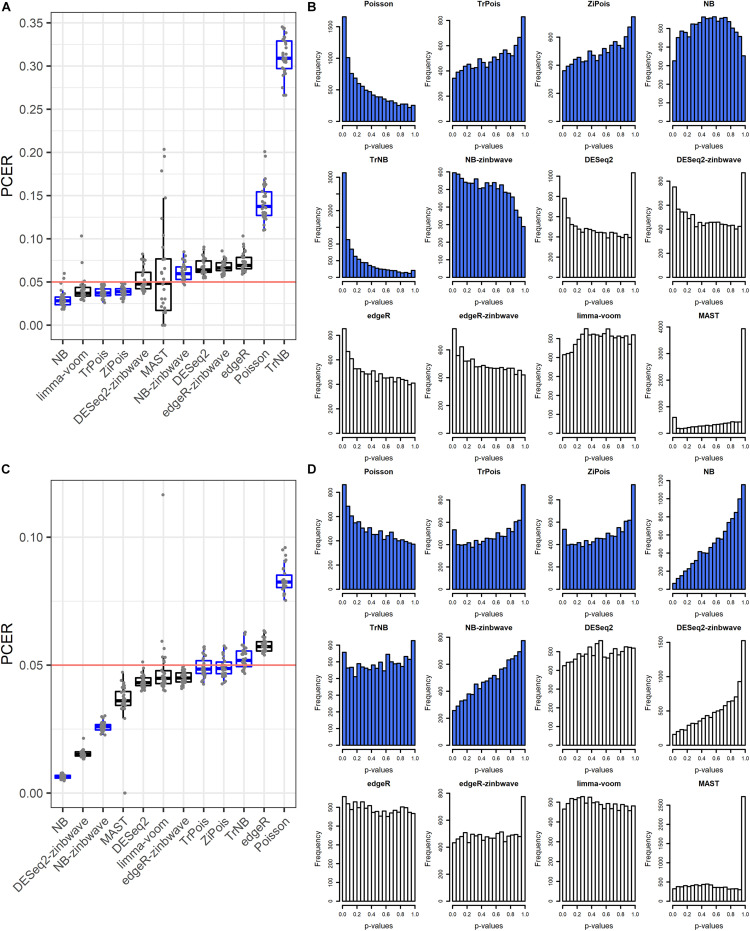
False positive rate control on simulated null Usoskin datasets and Tung datasets. **(A)** Boxplot of PCER for 30 simulated null Usoskin datasets generated by *splatter* for each of 12 DE methods. scMMSTs are marked in blue. **(B)** Histogram of uncorrected *p*-values for one dataset in panel **A**. **(C)** Boxplot of PCER for 30 simulated null Tung datasets generated by *splatter* for each of 12 DE methods. scMMSTs are marked in blue. **(D)** Histogram of uncorrected *p*-values for one dataset in panel **C**. PCER, per-comparison error rate; DE, differential expression; scMMST, single cell mixed model score test.

The FPR control was assessed by the PCER. Results are shown in Figure 1. For the Usoskin dataset, the estimated common dispersion parameter value of biological coefficient of variation (BCV) was $\hat{\phi }=1/\hat{\theta }=$1.89. TrNB and Poisson failed to control the FPR. The PCERs of NB-zinb, DESeq2, edgeR-zinb, and edgeR were a little inflated. DESeq2-zinb and MAST controlled the FPRs with large variability, especially for MAST. Other methods were a little conservative with PCERs smaller than the nominal level 0.05. For the Tung dataset, the estimated common dispersion parameter value of BCV was $\hat{\phi }=1/\hat{\theta }=0.11$. Poisson failed to control the FPR. The PCERs of TrNB and edgeR were a little inflated. Other methods conservatively controlled FPRs, especially for NB, DESeq2-zinb, and NB-zinb. We treated “NA” *p*-values of DE methods as 1, thus, there are peak bars at 1 for some methods in the unadjusted *p*-value histograms shown in Figures 1B,D. In summary, standard DE methods can control the FPRs and scMMSTs except Poisson and TrNB can conservatively control the FPRs. FPRs of scMMSTs increase as the dispersion parameter θ decreases.

**FIGURE 2:**
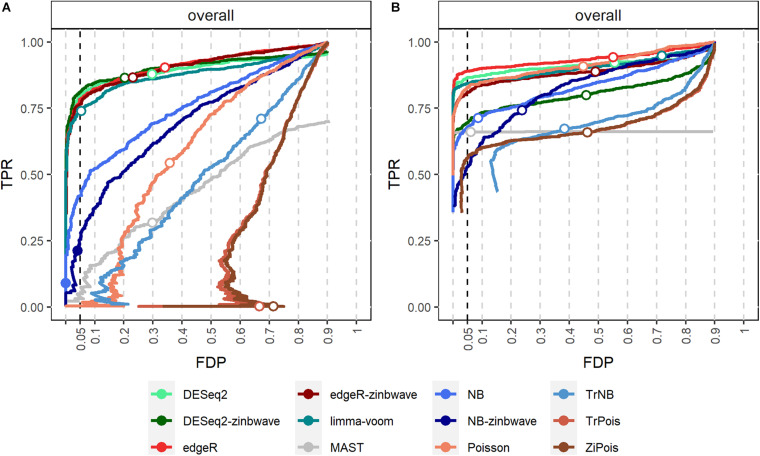
FDP-TPR curves of DE methods on simulated Usoskin datasets and Tung datasets. **(A)** Line plot of the FDP-TPR curves for simulated Usoskin datasets generated by *splatter* for each of 12 DE methods. **(B)** Line plot of the FDP-TPR curves for simulated Tang datasets generated by *splatter* for each of 12 DE methods. Circles represent values at a 0.05 nominal FDR threshold and are filled in if the FDP (i.e., empirical FDR) is less than 0.05. DE, differential expression; TPR, true positive rate; FDP, false discovery proportion; FDR, false discovery rate.

False discovery proportion-true positive rate curves for adjusted *p*-values are shown in Figure 2. For the Usoskin dataset, bulk RNA-seq DE methods are shown to perform well, possibly due to the high proportion of zeros and low counts (Van den Berge et al., 2018). In general, standard DE methods except MAST perform better than scMMSTs when the batch effects is not associated with group effects.

### Simulations by GLMMs

**FIGURE 3:**
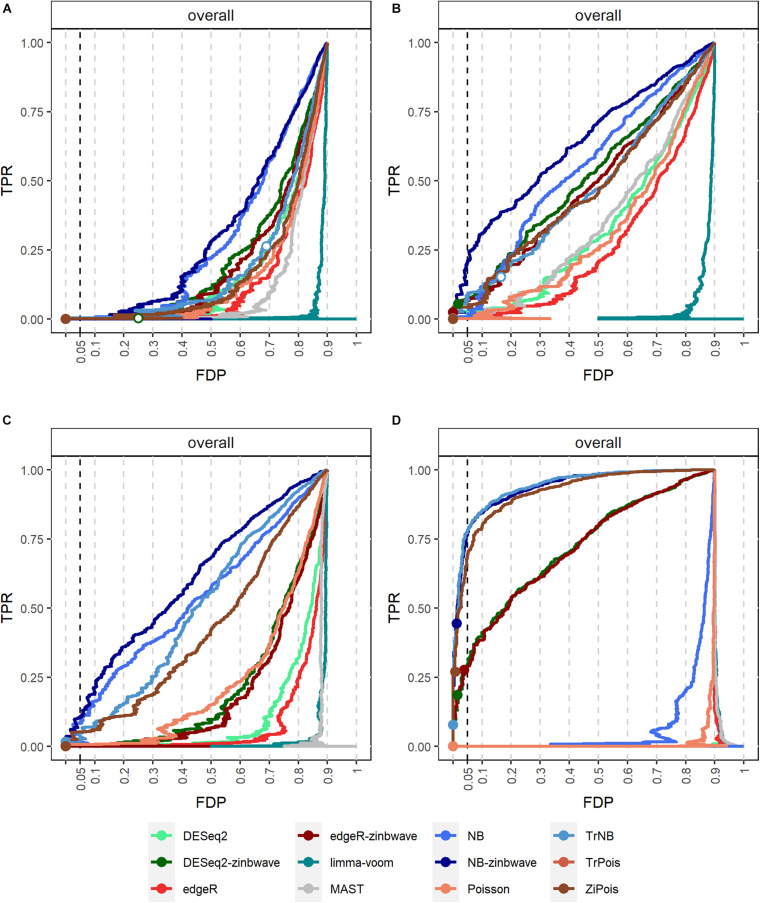
FDP-TPR curves of DE methods on simulated datasets generated by GLMMs with μ_π_ = 0. **(A)** Line plot of the FDP-TPR curves for simulated datasets based on NB GLMMs for each of 12 DE methods with the dispersion parameter θ = 0.5. **(B)** Line plot of the FDP-TPR curves for simulated datasets based on negative binomial (NB) GLMMs for each of 12 DE methods with θ = 1. **(C)** Line plot of the FDP-TPR curves for simulated datasets based on NB GLMMs for each of 12 DE methods with θ = 2. **(D)** Line plot of the FDP-TPR curves for simulated datasets based on Poisson GLMMs for each of 12 DE methods with $\beta_{0}=\sigma_{\beta }^{2}=$ 0.01. Circles represent values at a 0.05 nominal FDR threshold and are filled in if the FDP (i.e., empirical FDR) is less than 0.05. DE, differential expression; GLMM, generalized linear mixed model; NB, negative binomial; TPR, true positive rate; FDP, false discovery proportion; FDR, false discovery rate.

**FIGURE 4:**
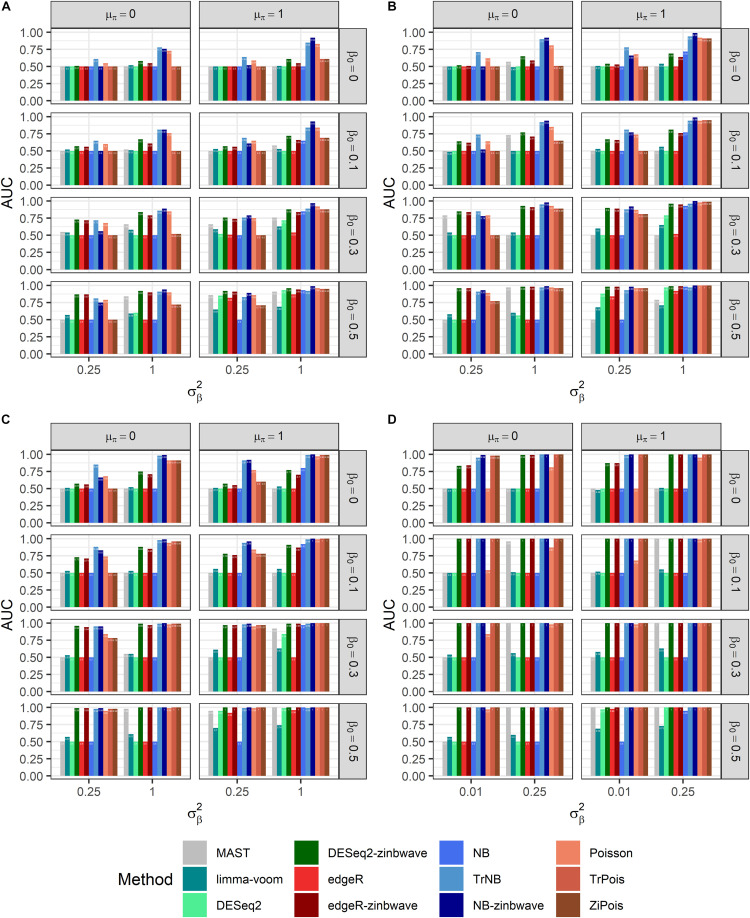
AUCs of DE methods for simulated datasets generated by GLMMs with μ_π_ = 0. Adjusted *p*-values are used as predictors. **(A)** Bar plot of AUCs for simulated datasets generated by NB GLMMs for each of 12 DE methods with the dispersion parameter θ = 0.5. **(B)** Bar plot of AUCs for simulated datasets generated by NB GLMMs for each of 12 DE methods with θ = 1. **(C)** Bar plot of AUCs for simulated datasets generated by NB GLMMs for each of 12 DE methods with θ = 2. **(D)** Bar plot of AUCs for simulated datasets generated by Poisson GLMMs for each of 12 DE methods. AUC, area under curve; DE, differential expression; GLMM, generalized linear mixed model; NB, negative binomial.

Results of PCERs are shown in Supplementary Figures S2, S3 and Supplementary Table S1. Methods performances of the FPR control were similar to those in simulations by *splatter*. Based on FDP-TPR curves for adjusted *p*-values shown in Figure 3, scMMSTs performed better than standard DE methods when batch effects were associated with weak group effects. NB-zinb was the best among all methods considered for comparisons. EdgeR-zinb and DESeq2-zinb were the best two methods among the six standard DE methods considered. TrPois and ZiPois perform very close to each other. Figure 4 demonstrates bar plots of AUCs for adjusted *p*-values. |β_0_|, $\sigma_{\beta }^{2}$, θ and μ_π_ exhibited positive correlations with AUCs. Our scMMSTs performed better when the group effect size and its heterogeneity are larger and the counts dispersion BCV and proportion of zeros are smaller. Similar results are obtained to those of FDP-TPR curves. Therefore, our results demonstrate that scMMSTs performs better than standard DE methods when the group effect size is small with large group effect heterogeneity.

### Real Data Analysis

Table 1 and Supplementary Figure S4 show the numbers of DE genes detected by the 12 methods considered in simulations for 11 cell types in the Usoskin dataset. This dataset was also analyzed in the *zinbwave* paper. MAST failed for some cell-types, so no DE gene was detected. NB-zinb defined smallest number of DE genes in general. The results of Venn diagrams and Upset plots by R packages *VennDiagram* (version 1.6.20) (Chen, 2018) and *upsetR* (version 1.4.0) (Gehlenborg, 2019) are shown in Supplementary Figures S5–S15. Since NB-zinb is conservative for FDR, the DE genes only detected by NB-zinb highly likely have weak group effects with their heterogeneity across batches. In general, scMMSTs, as supplement to standard methods, are superior at selecting DE genes with weak group effects and their heterogeneity in different batches for scRNA-seq data.

**TABLE 1:** Numbers of declared differentially expressed genes by 12 methods for 11 defined cell types vs. the rest in the Usoskin dataset (*n* = 622 cells).

**Methods**	**NF1**	**NF2**	**NF3**	**NF4**	**NF5**	**NP1**	**NP2**	**NP3**	**PEP1**	**PEP2**	**TH**
edgeR	826	1206	348	646	1070	1877	880	362	1833	328	2424
DESeq2	906	963	218	402	782	1988	748	407	2649	102	2387
limma-voom	5427	3762	3777	721	2572	2505	4857	203	7892	173	4800
MAST	0	0	0	2	0	85	5	2	10	0	112
edgeR-zinb	509	778	244	550	985	1871	987	486	2475	185	3225
DESeq2-zinb	555	1003	319	453	1235	1985	786	392	2249	153	3166
NB	295	517	186	365	555	462	329	218	592	145	533
TrNB	910	703	596	1763	885	1127	2139	2254	3752	537	1986
NB-zinb	192	308	77	295	364	976	467	270	2004	100	878
Pois	745	1214	410	881	1195	1401	745	583	2104	339	1942
TrPois	242	298	82	345	321	602	756	444	3353	54	708
ZiPois	337	311	81	487	376	607	1019	446	3350	137	704

### Computational Time

**FIGURE 5:**
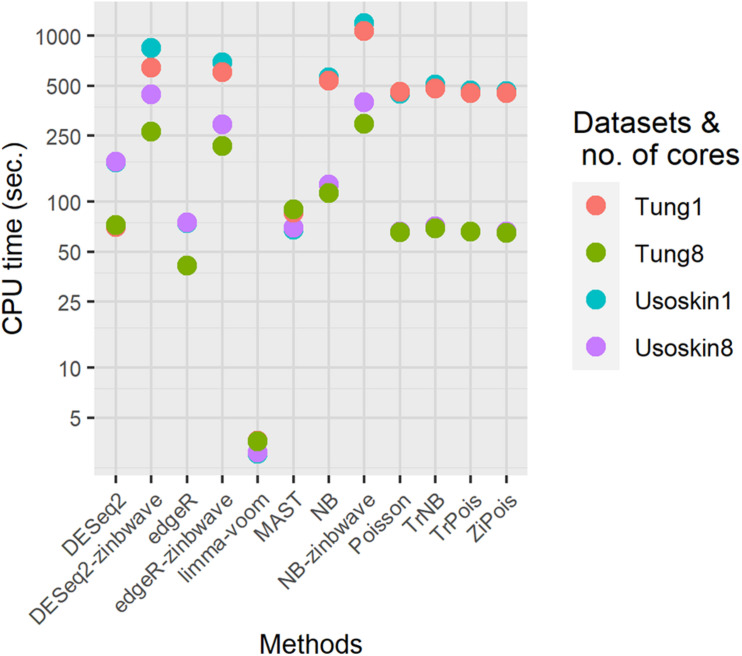
Computational times for differential expression methods on the simulated null Usoskin and Tung datasets, which were generated by *splatter*. The number of cores were set to be 1 and 8 on a cluster with 24 Intel Xeon Processor (Skylake, IBRS) at 2.60 GHz (2593 MHz) and 128 GB RAM.

To demonstrate the computation time scale of DE methods considered, we benchmarked two different simulated null datasets by *splatter* with parameters estimated by the Usoskin and Tung datasets. Other settings remained the same as those in the simulations for PCERs. Results are shown in Figure 5. For both datasets, the fastest method was limma-voom. DESeq2 was slower than edgR, thus, DESeq2-zinb was also slower than edgeR-zinb. Our scMMSTs performed in the same scale of DESeq2-zinb and DESeq2-zinb. The computation times of simulated null Tung datasets were shorter than those of simulated null Usoskin datasets with the same number of cores. More cores used in the parallel computation made our scMMSTs faster. With eight cores, the computation times of Poisson related methods were close to MAST, edgeR, and DESeq2. In summary, our scMMSTs are computationally affordable compared to other DE methods especially when parallel computing is allowed. All computations were done on a cluster with 24 Intel Xeon Processor (Skylake, IBRS) at 2.60 GHz (2593 MHz) and 128 GB RAM.

## Discussion

We proposed scMMSTs to identify DE genes, considering batch effect and zero inflation of scRNA-seq data. Both simulations and real data indicated that these methods have advantages in selecting DE genes with weak group effects and their heterogeneity in different batches. In simulations, scMMSTs conservatively controlled FPRs or type I error rates in each setting under assumptions of NB and Poisson distributions, except TrNB and Poisson assumption. However, TrNB controlled FPRs when θ is large. Second, following the model assumption, scMMST was the best one when |β_0_| was small and $\sigma_{\beta }^{2}$ was large, especially when θ was large. In real data analysis, the Venn diagrams and Upset plots of DE genes (Supplementary Figures S5–S15) directly indicated the relationships among the DE methods. scMMATs defined smaller numbers of DE genes and NB-zinb defined the smallest. Since scMMATs are conservative, the DE genes only defined by NB-zinb are likely to have the small group effect size with its heterogeneity across batches.

Furthermore, scMMSTs exhibited three innovations. First, scMMSTs derived the association test score statistics and their theoretical null distributions in the framework of GLMMs under the binomial, Poisson and NB assumptions. Second, the group effect β was modeled as random effects associated with batches in the framework of GLMMs. Third, scMMSTs verified their effectiveness to detect DE genes with the weak group effect and its heterogeneity in different batches. However, scMMSTs have some limitations. scMMSTs performed worse than other standard DE methods to detect DE genes without group effect heterogeneity across batches. scMMSTs performed worse when the dispersion parameter θ was small, especially for the TrNB method, this may due to the non-robust estimation of θ. scMMSTs, in fact, are derived to test $H_{0}^{\prime }$under the assumption β_0_ = 0, not to jointly test β_0_ = 0 and $\sigma_{\beta }^{2}=0$. This decreases the power of testing *H_0* for scMMSTs. For association tests, the Mixed effects Score Test (MiST), which jointly tests *H*_0_, is more powerful. Therefore, scMMSTs may be extended using the framework of GLMM-MiST (Sun et al., 2013) in future work to overcome these drawbacks. ${\text{E}}_{w}^{\prime }$ is used to approximate E_**w**_ for the statistic **T_w_** of scMMSTs. This approximation performs worse when there are more excess zeros. Better approximations of E**_w_** or methods to efficiently calculate E**_w_** may improve the performance of scMMSTs. The weighting strategy implemented may be explained in a Bayesian framework and scMMSTs may be extended accordingly. In addition, following the idea of PEA (Shao et al., 2019), scMMSTs may be extended to efficiently identify gene-pathway interactions without permutations of test statistics. In conclusion, scMMSTs, supplements to standard single cell DE methods, are advantageous at selecting genes with the weak group effect and its heterogeneity across batches for scRNA-seq data analysis.

## Data Availability Statement

Publicly available datasets were analyzed in this study. These data can be found here: the dataset (Usoskin) analyzed for this study can be found in the [Github respiratory of the *zinbwave* paper (Van den Berge et al., 2018)] (https://github.com/statOmics/zinbwaveZinger/blob/master/datasets/esetUsoskin.RData); the dataset (Tung) can be found in the [Github respiratory of the splatter paper (Zappia et al., 2017)](https://github.com/Oshlack/splatter-paper/blob/master/data.tar.gz).

## Author Contributions

FS and HW conceived and supervised the study. ZH and FS implemented the software, conducted the simulations, analyzed the data, and wrote the manuscript. ZH and YP prepared figures and tables. ZH, YP, HW, and FS modified and reviewed the manuscript. All authors contributed to the article and approved the submitted version.

## Conflict of Interest

The authors declare that the research was conducted in the absence of any commercial or financial relationships that could be construed as a potential conflict of interest.

## Footnotes

1 https://github.com/biostat0903/RNAseq-Data-Analysis

2 https://github.com/statOmics/zinbwaveZinger

3 https://github.com/statOmics/zinbwaveZinger/tree/master/datasets

4 https://github.com/jdblischak/singleCellSeq

5 https://github.com/Oshlack/splatter-paper
