# Supplementary material for: Identifying Differentially Expressed Genes of Zero Inflated Single Cell RNA Sequencing Data Using Mixed Model Score Tests
Source: Front Genet. 2021 Feb 5;12:616686. doi: 10.3389/fgene.2021.616686 (PMC7894898; doi:10.3389/fgene.2021.616686)
Supplement: Supplementary file 2 [file Data_Sheet_2.docx]

Supplementary Material

# Supplementary Data

**Data S1.** R code for implementing scMMSTs and reproducing all analyses and figures reported in the manuscript.

# Supplementary Figures and Tables

## Supplementary Figures

**Figure S1.** Demonstration of associations of sequencing depth and zero inflation for the Usoskin dataset and histograms of observational weights by *zinbwave* for the Usoskin and Tung datasets. **(A)** The scatter plot and logistic regression fits with picking sessions as the batches for the Usoskin dataset. This is the repeat of Fig. 5a of the *zinbwave* paper. **(B)** Histogram of observational weights for nonzero counts in the Usoskin dataset calculated by the ZINB-WaVE model including the cell type as a covariate with and without the batch effect as fixed effects. This is the repeat of Fig. 5b of the *zinbwave* paper. **(C)** Histogram of observational weights for nonzero counts in the filtered Tung dataset (18726 genes with more than 0 count) calculated by the ZINB-WaVE model. **(D)** Histogram of observational weights for nonzero counts in the filtered Tung dataset (14893 genes with more than 19 counts) calculated by the ZINB-WaVE model.

**Figure S2.** False positive control on simulated null GLIMM datasets with $\mu_{\pi}$=0. **(A)** Boxplot of PCER for 30 simulated null datasets generated by NB GLIMM with *θ*=0.5 for each of twelve DE methods. scMMSTs are highlighted in blue. **(B)** Histogram of unadjusted p-values for one of the datasets in **A**. **(C)** Boxplot of PCER for 30 simulated null Tung datasets generated by NB GLIMM with *θ*=1 for each of twelve DE methods. scMMSTs are highlighted in blue. **(D)** Histogram of unadjusted p-values for one of the datasets in **C**. GLMM generalized linear mixed model, PCER per-comparison error rate, NB negative binomial, DE differential expression, scMMST single cell mixed model score test.

**Figure S3.** False positive control on simulated null GLIMM datasets with $\mu_{\pi}$=0. **(A)** Boxplot of PCER for 30 simulated null datasets generated by NB GLIMM with *θ*=2 for each of twelve DE methods. scMMSTs are highlighted in blue. **(B)** Histogram of unadjusted p-values for one of the datasets in **A**. **(C)** Boxplot of PCER for 30 simulated null Tung datasets generated by Poisson GLIMM for each of twelve DE methods. scMMSTs are highlighted in blue. **(D)** Histogram of unadjusted p-values for one of the datasets in **C**. GLMM generalized linear mixed model, PCER per-comparison error rate, NB negative binomial, DE differential expression, scMMST single cell mixed model score test.

**Figure S4.** The number of differentially expressed genes identified by the twelve DE methods considered in simulations for the Usoskin dataset (n=622 cells).

**Figure S5.** Results for differential expression methods on the cell type NF1 vs. the rest of the Usoskin dataset (n=622 cells). **(A)** Venn diagram. **(B)** Upset plot.

**Figure S6.** Results for differential expression methods on the cell type NF2 vs. the rest of the Usoskin dataset (n=622 cells). **(A)** Venn diagram. **(B)** Upset plot.

**Figure S7.** Results for differential expression methods on the cell type NF3 vs. the rest of the Usoskin dataset (n=622 cells). **(A)** Venn diagram. **(B)** Upset plot.

**Figure S8.** Results for differential expression methods on the cell type NF4 vs. the rest of the Usoskin dataset (n=622 cells). **(A)** Venn diagram. **(B)** Upset plot.

**Figure S9.** Results for differential expression methods on the cell type NF5 vs. the rest of the Usoskin dataset (n=622 cells). **(A)** Venn diagram. **(B)** Upset plot.

**Figure S10.** Results for differential expression methods on the cell type NP1 vs. the rest of the Usoskin dataset (n=622 cells). **(A)** Venn diagram. **(B)** Upset plot.

**Figure S11.** Results for differential expression methods on the cell type NP2 vs. the rest of the Usoskin dataset (n=622 cells). **(A)** Venn diagram. **(B)** Upset plot.

**Figure S12.** Results for differential expression methods on the cell type NP3 vs. the rest of the Usoskin dataset (n=622 cells). **(A)** Venn diagram. **(B)** Upset plot.

**Figure S13.** Results for differential expression methods on the cell type PEP1 vs. the rest of the Usoskin dataset (n=622 cells). **(A)** Venn diagram. **(B)** Upset plot.

**Figure S14.** Results for differential expression methods on the cell type NEP2 vs. the rest of the Usoskin dataset (n=622 cells). **(A)** Venn diagram. **(B)** Upset plot.

**Figure S15.** Results for differential expression methods on the cell type TH vs. the rest of the Usoskin dataset (n=622 cells). **(A)** Venn diagram. **(B)** Upset plot.

## Supplementary Tables

**Table S1.** False positive rate control of twelve differential expression methods with median per-comparison error rates and interquartile ranges (in parentheses) for 30 simulated null datasets. Simulated null Usoskin and Tung datasets are generated by *splatter* and simulated null negative binomial (NB) and Poisson datasets are generated by generalized linear mixed models.
