# Supplementary figures and images for: Identifying Differentially Expressed Genes of Zero Inflated Single Cell RNA Sequencing Data Using Mixed Model Score Tests
Source: Front Genet. 2021 Feb 5;12:616686. doi: 10.3389/fgene.2021.616686 (PMC7894898; doi:10.3389/fgene.2021.616686)

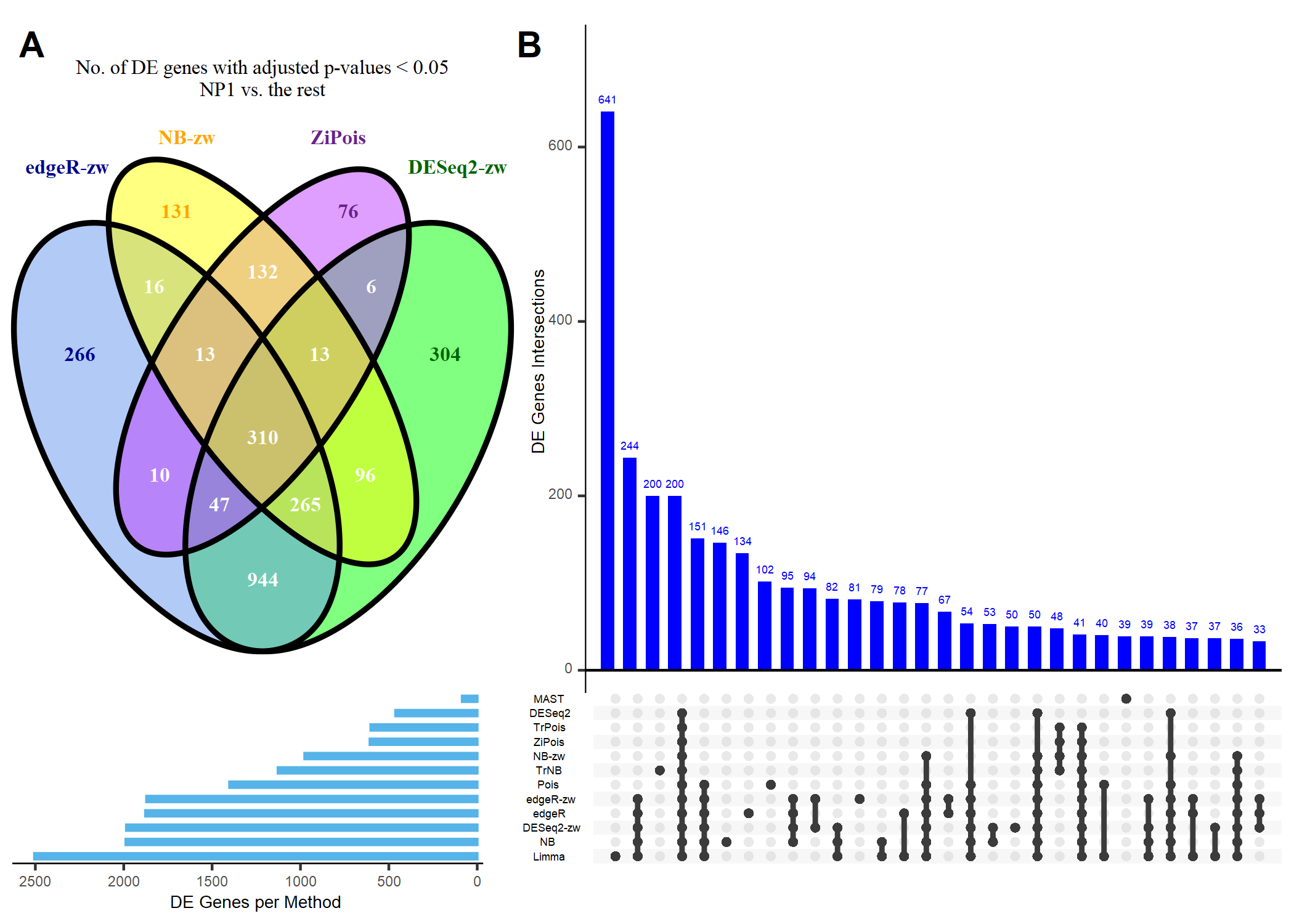

Supplement: Supplementary file 3 [file Presentation_1.zip › Supplementary_Figures/Figure_S10_Usoskin06NP1.tiff]

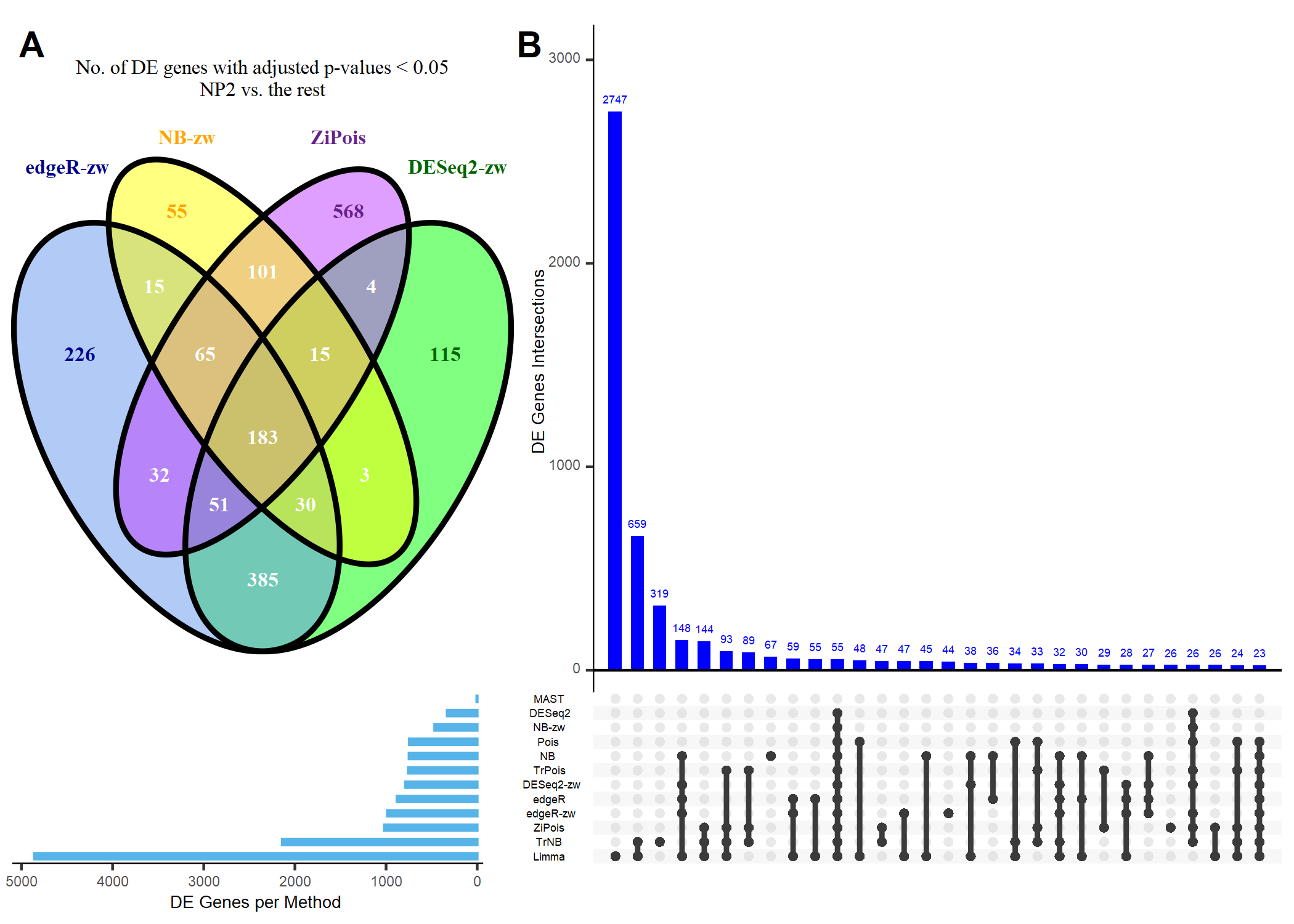

Supplement: Supplementary file 3 [file Presentation_1.zip › Supplementary_Figures/Figure_S11_Usoskin07NP2.tiff]

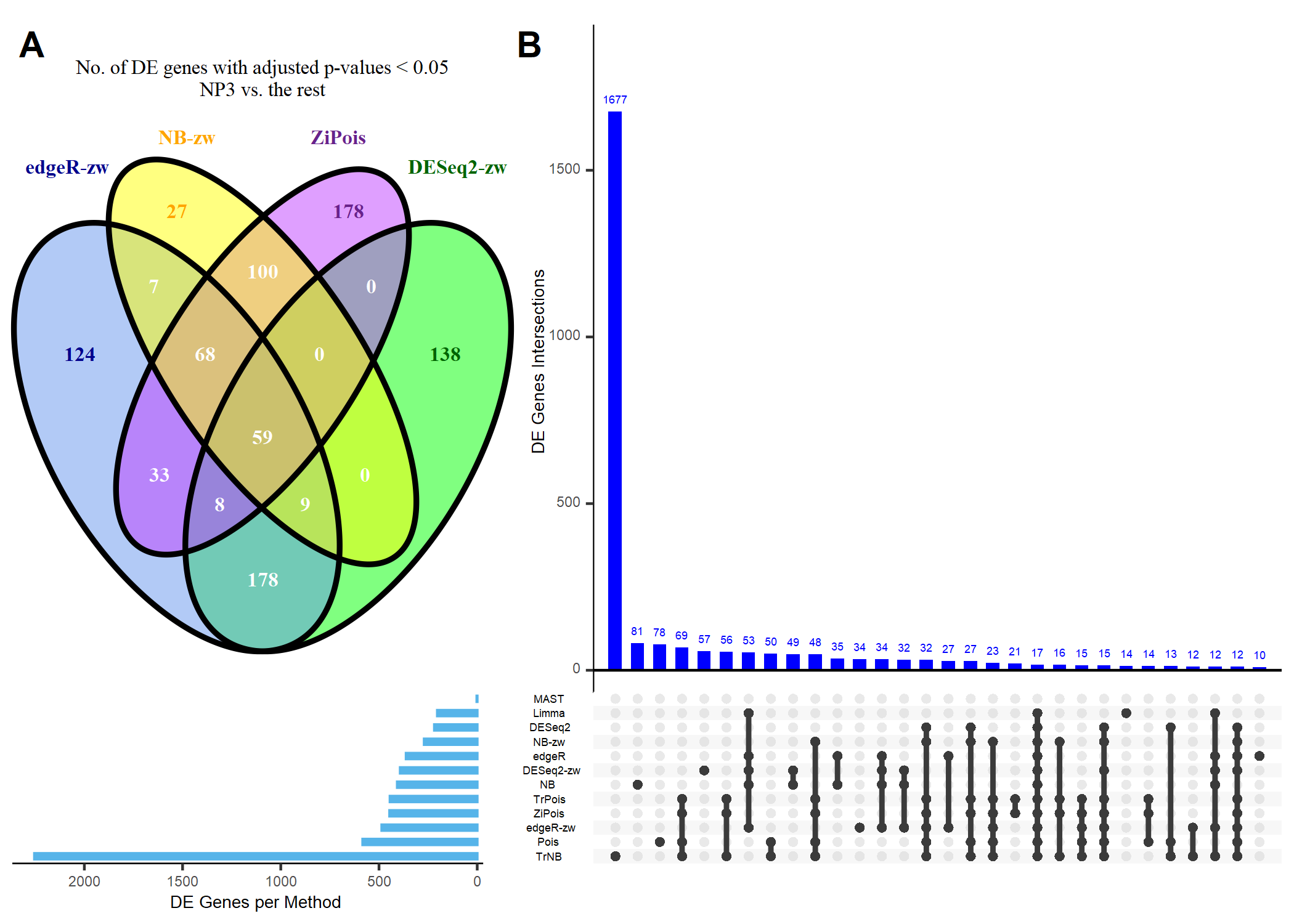

Supplement: Supplementary file 3 [file Presentation_1.zip › Supplementary_Figures/Figure_S12_Usoskin08NP3.tiff]

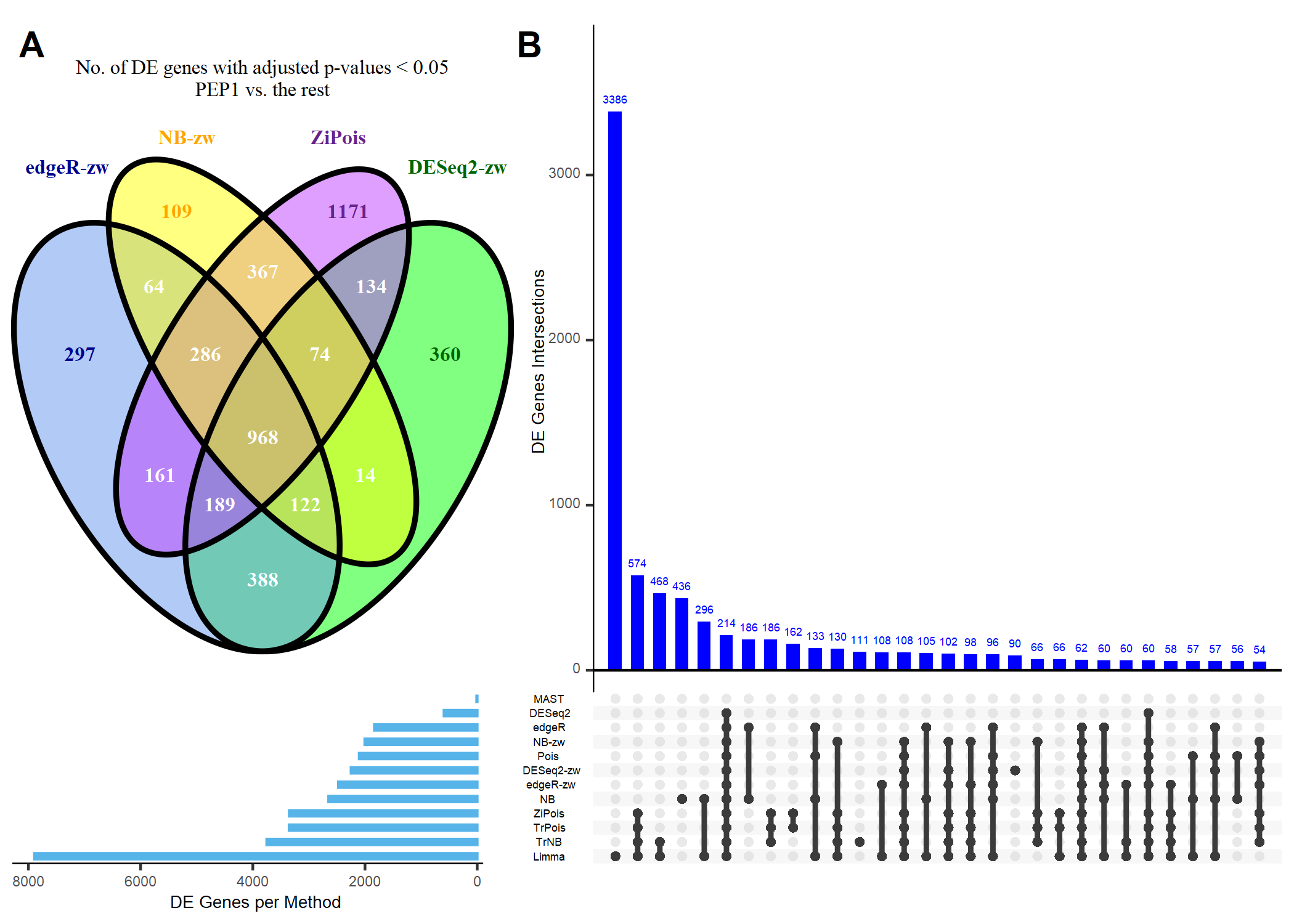

Supplement: Supplementary file 3 [file Presentation_1.zip › Supplementary_Figures/Figure_S13_Usoskin09PEP1.tiff]

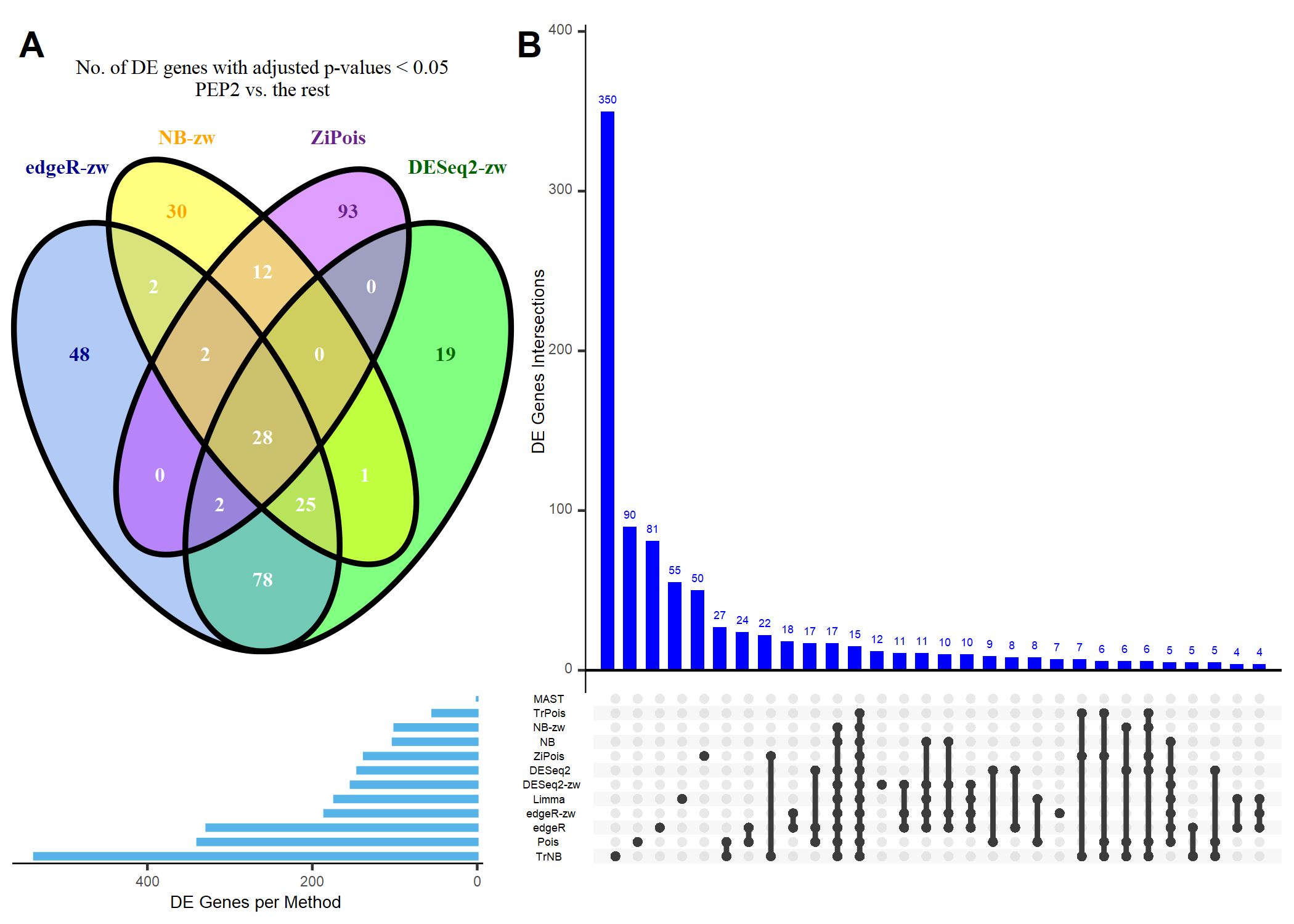

Supplement: Supplementary file 3 [file Presentation_1.zip › Supplementary_Figures/Figure_S14_Usoskin10PEP2.tiff]

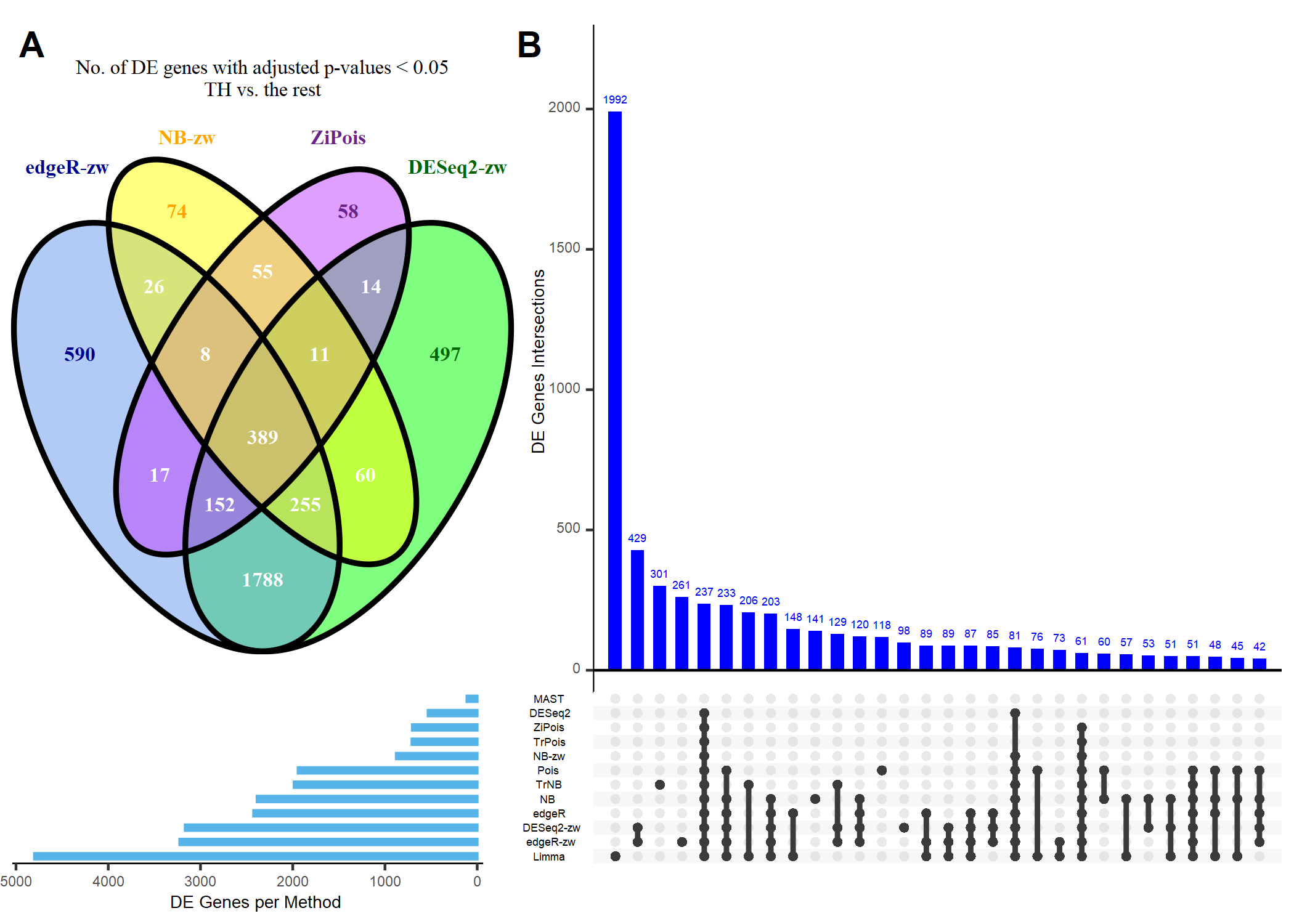

Supplement: Supplementary file 3 [file Presentation_1.zip › Supplementary_Figures/Figure_S15_Usoskin11TH.tiff]

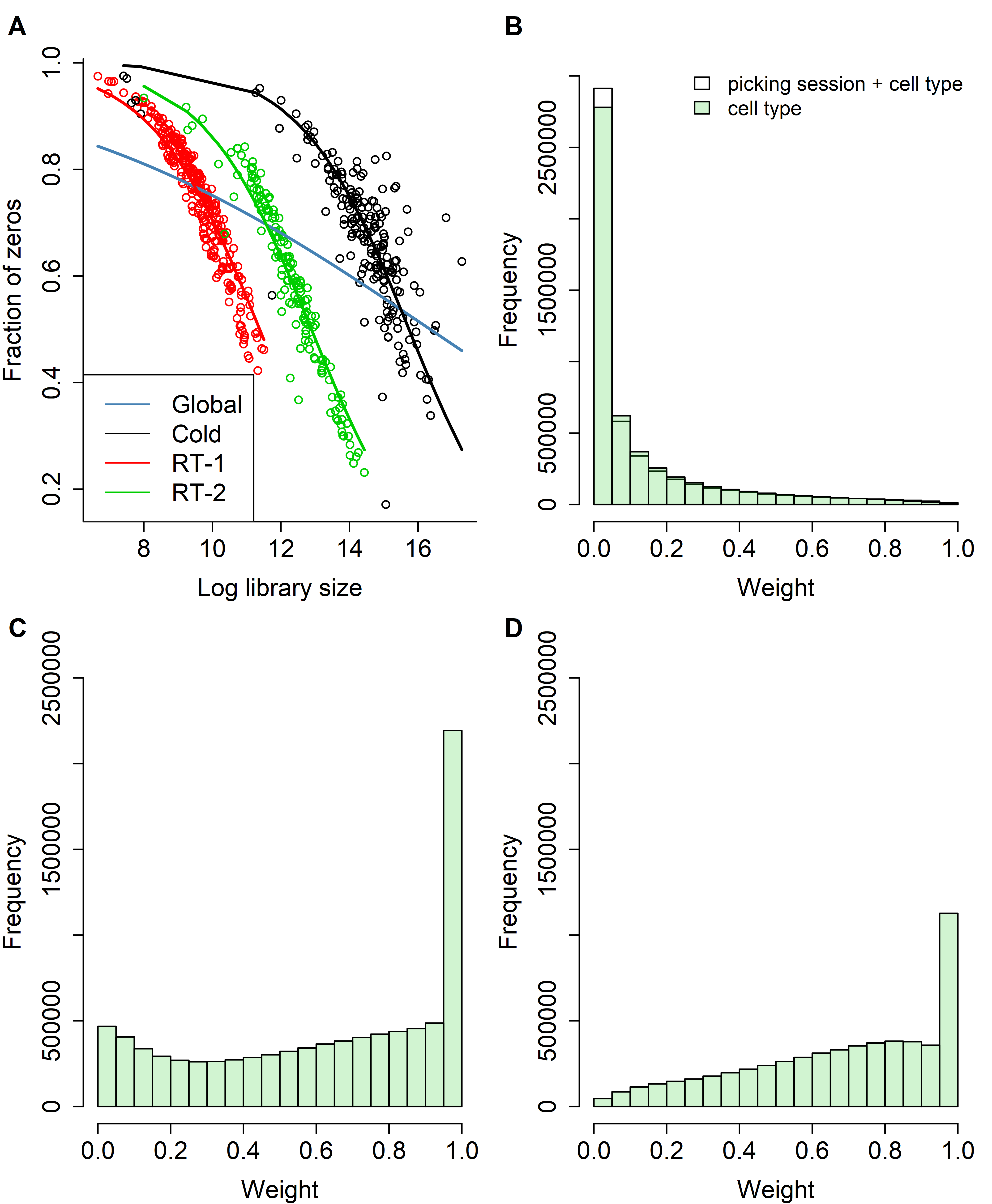

Supplement: Supplementary file 3 [file Presentation_1.zip › Supplementary_Figures/Figure_S1_Zero inflation.tiff]

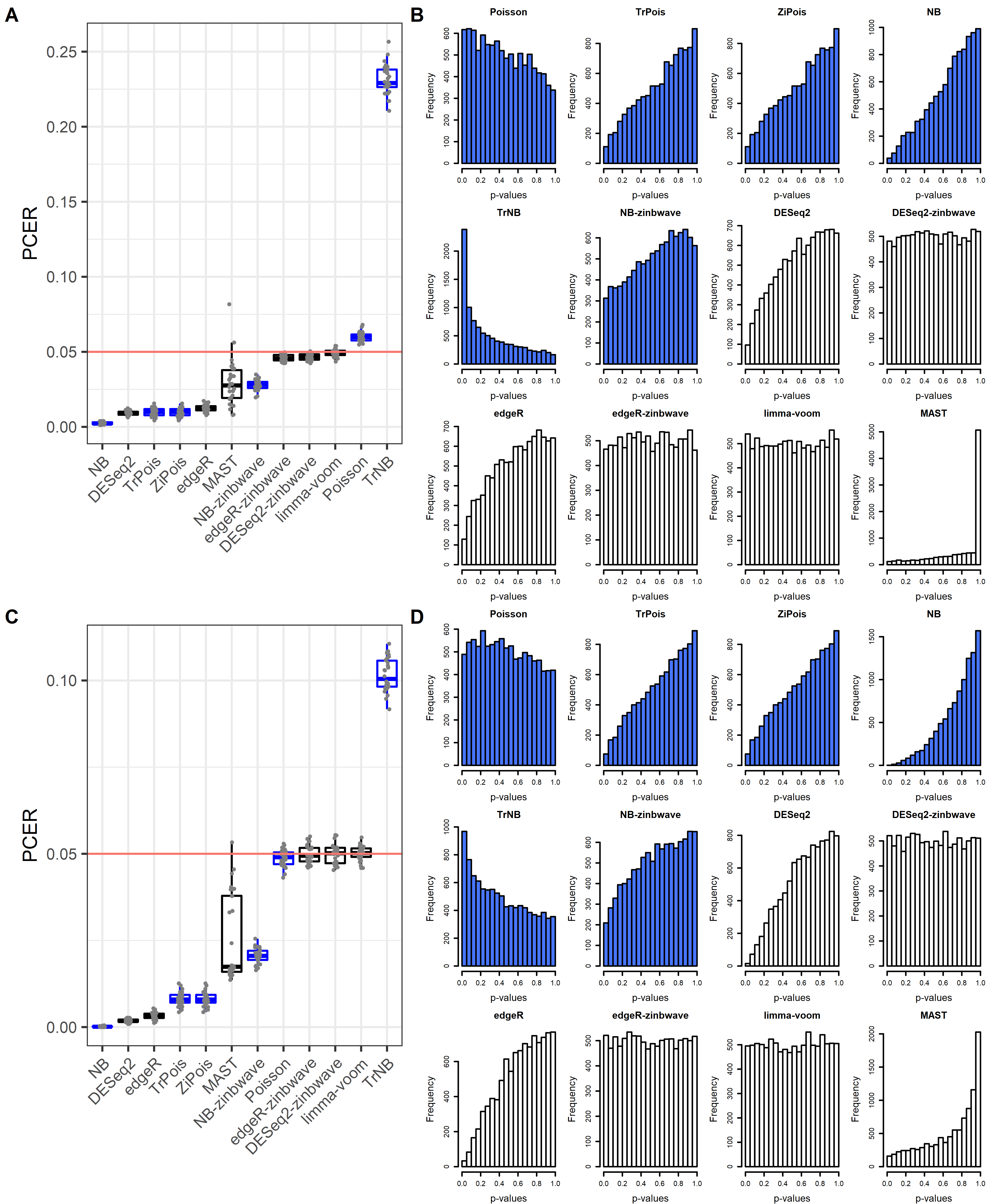

Supplement: Supplementary file 3 [file Presentation_1.zip › Supplementary_Figures/Figure_S2_FPR.tiff]

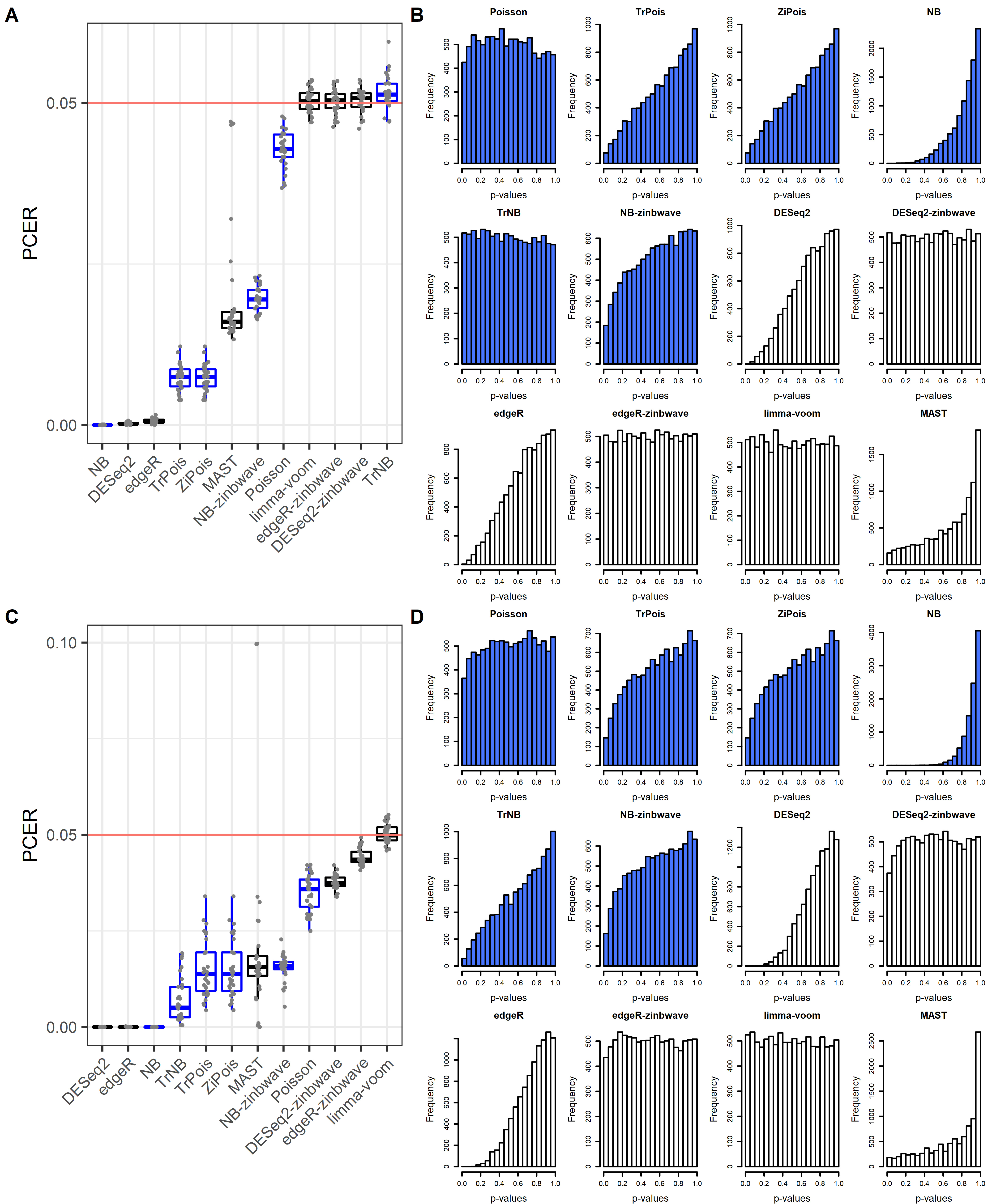

Supplement: Supplementary file 3 [file Presentation_1.zip › Supplementary_Figures/Figure_S3_FPR.tiff]

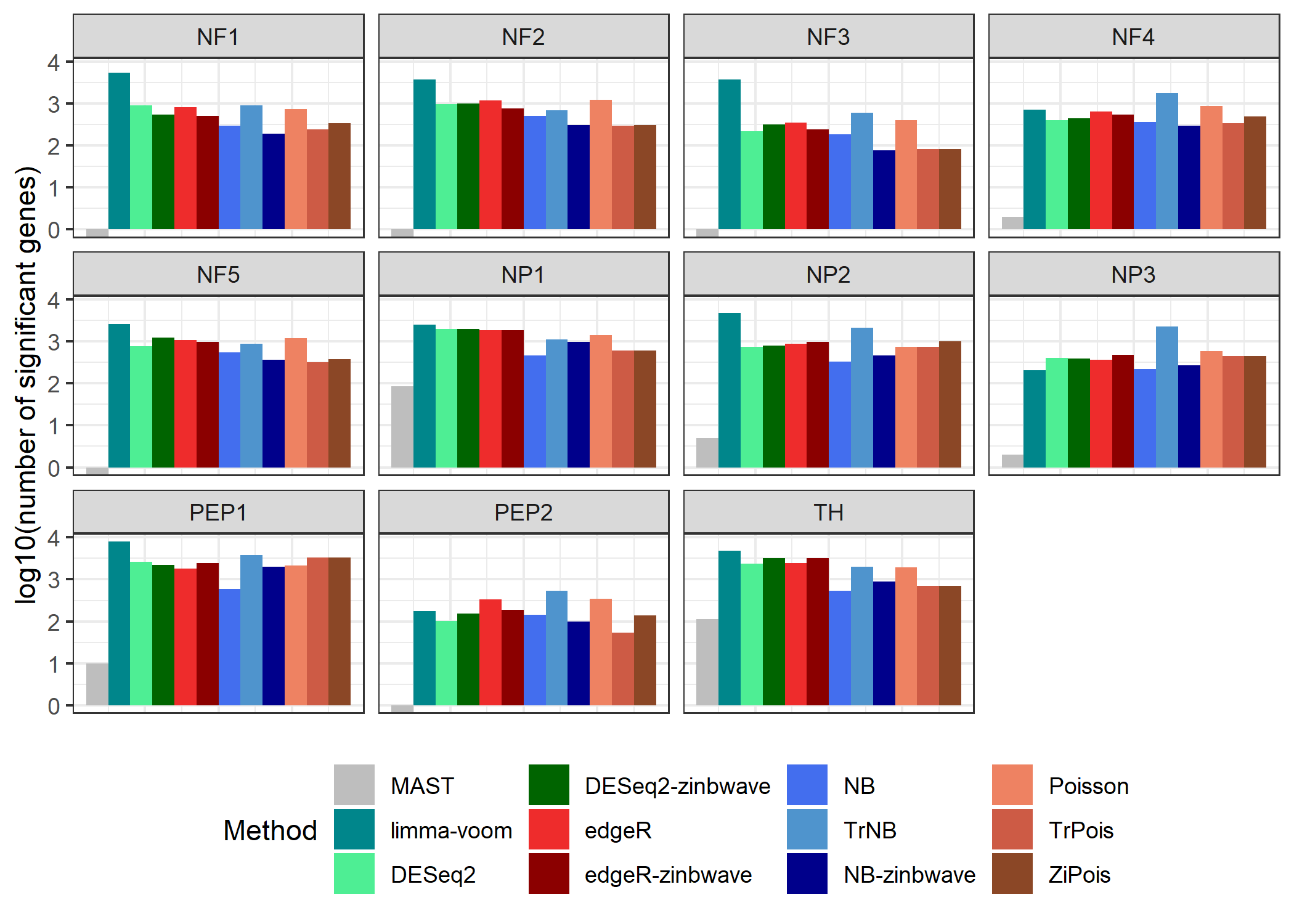

Supplement: Supplementary file 3 [file Presentation_1.zip › Supplementary_Figures/Figure_S4_DE genes #.tiff]

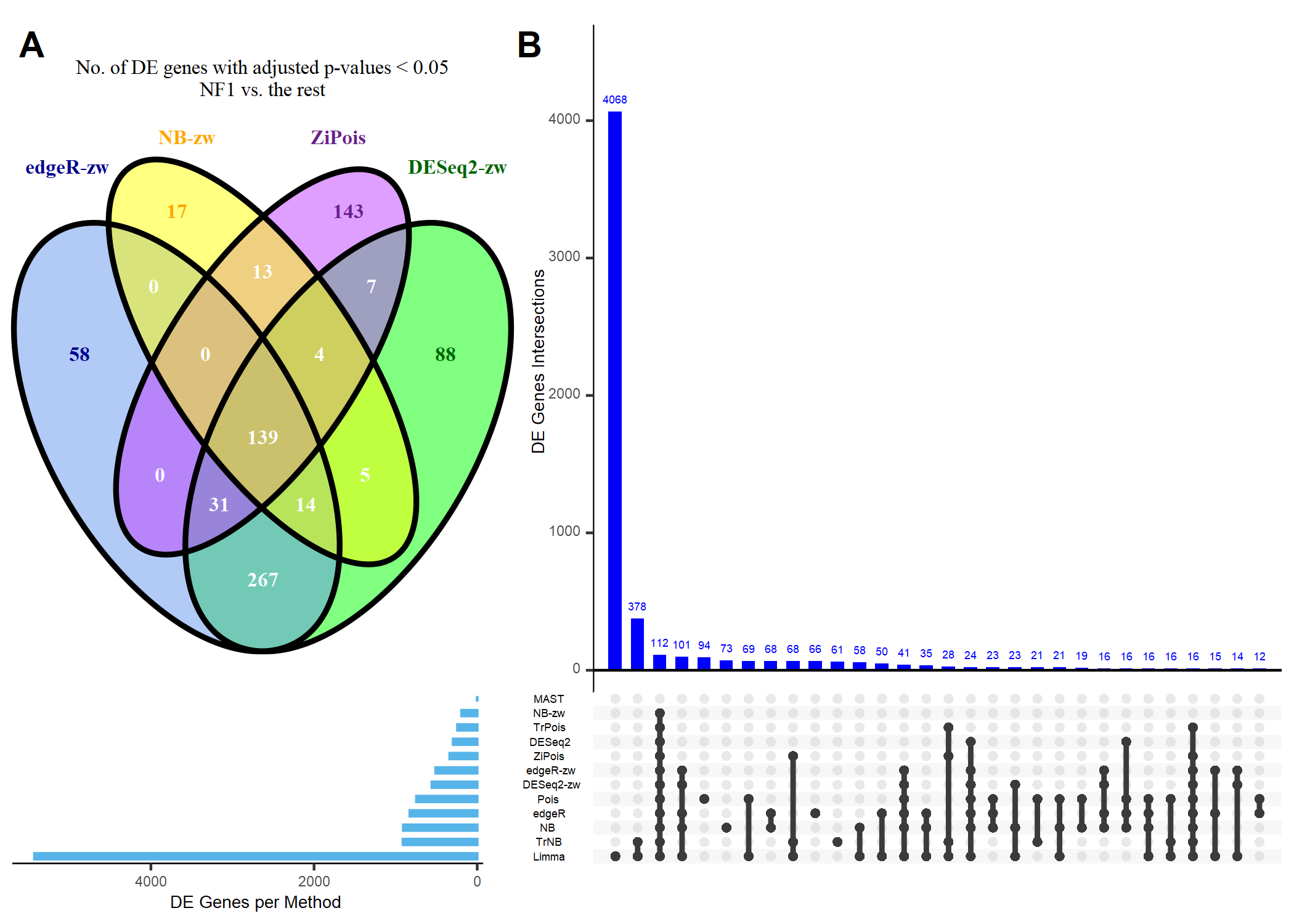

Supplement: Supplementary file 3 [file Presentation_1.zip › Supplementary_Figures/Figure_S5_Usoskin01NF1.tiff]

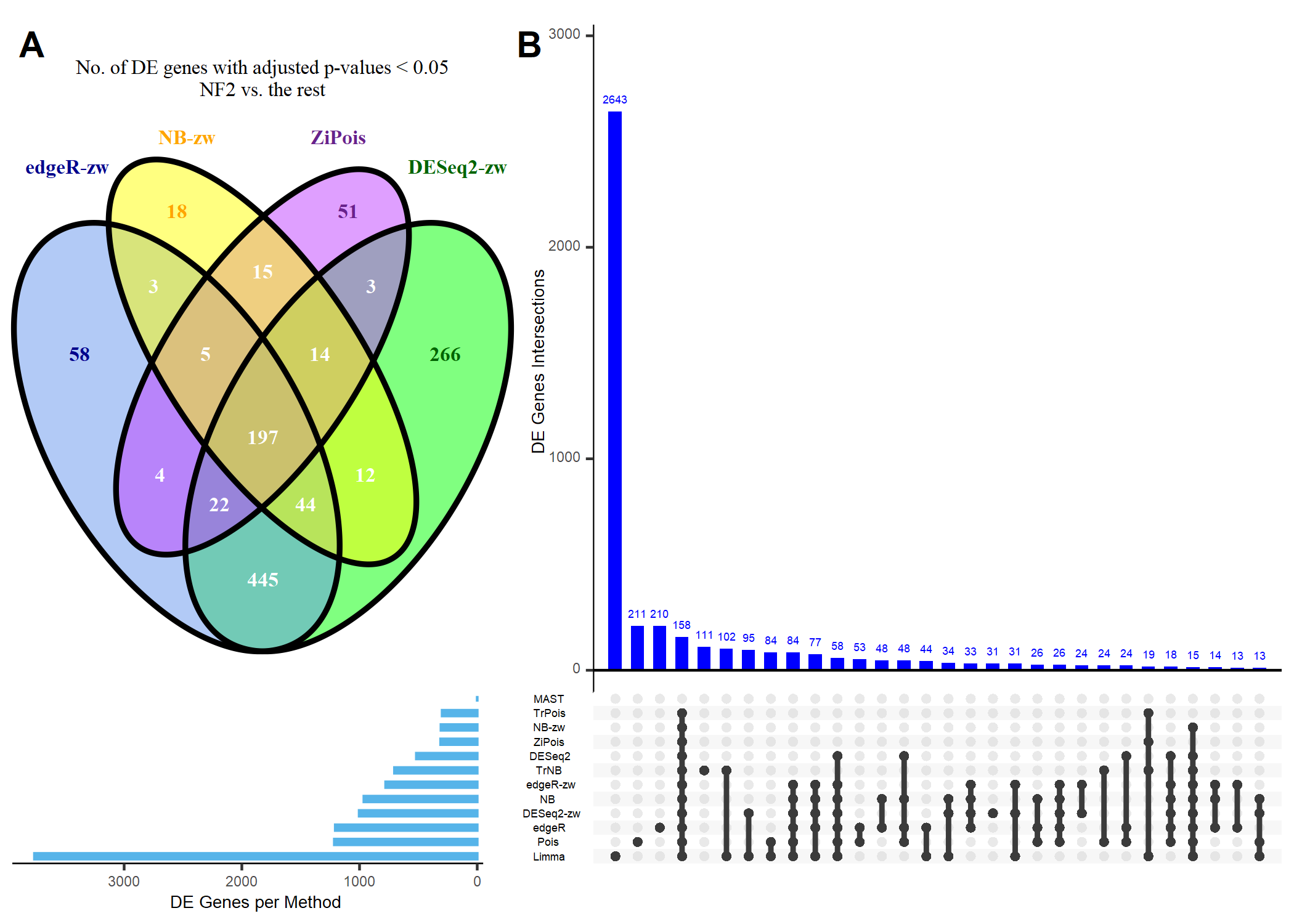

Supplement: Supplementary file 3 [file Presentation_1.zip › Supplementary_Figures/Figure_S6_Usoskin02NF2.tiff]

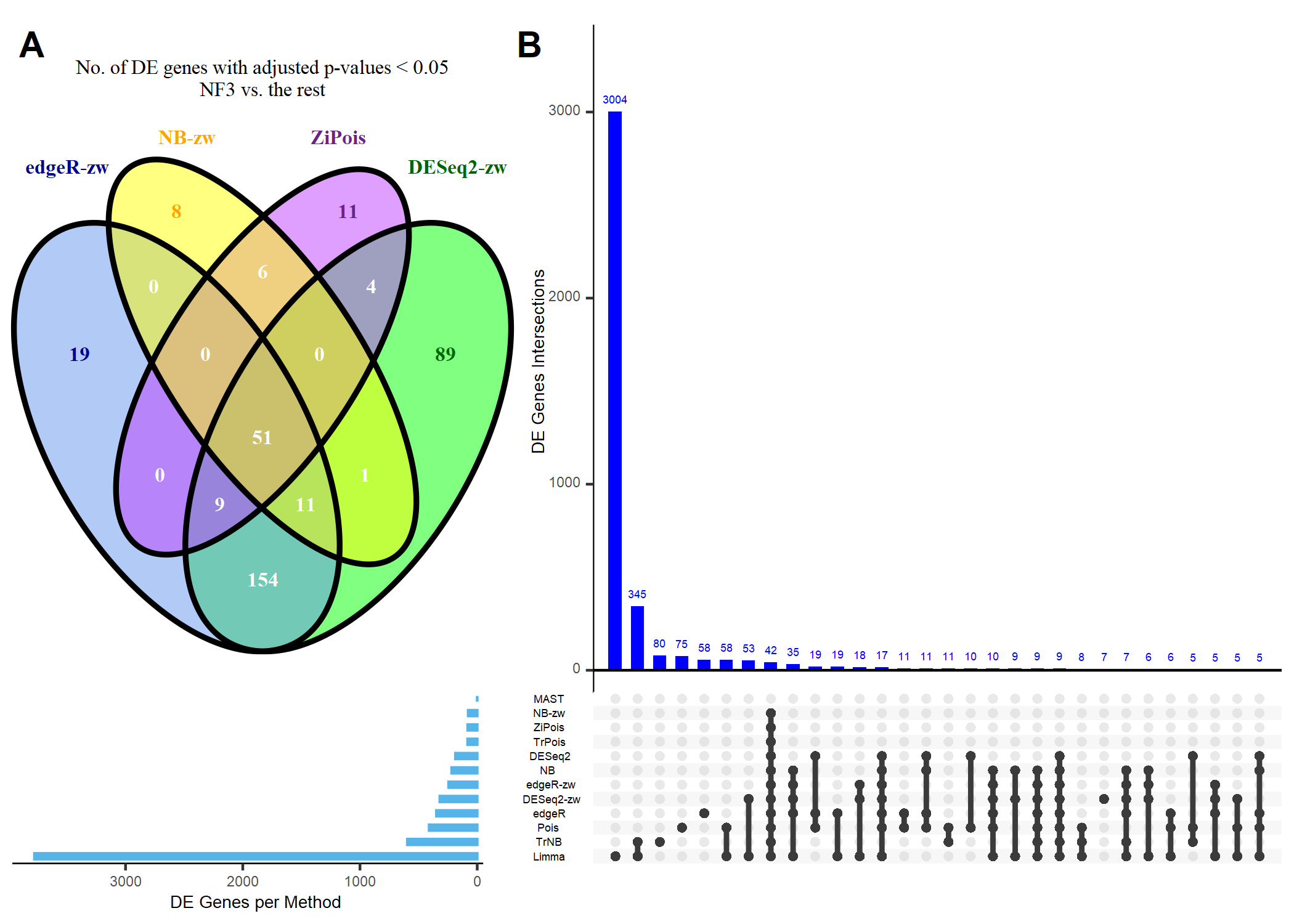

Supplement: Supplementary file 3 [file Presentation_1.zip › Supplementary_Figures/Figure_S7_Usoskin03NF3.tiff]

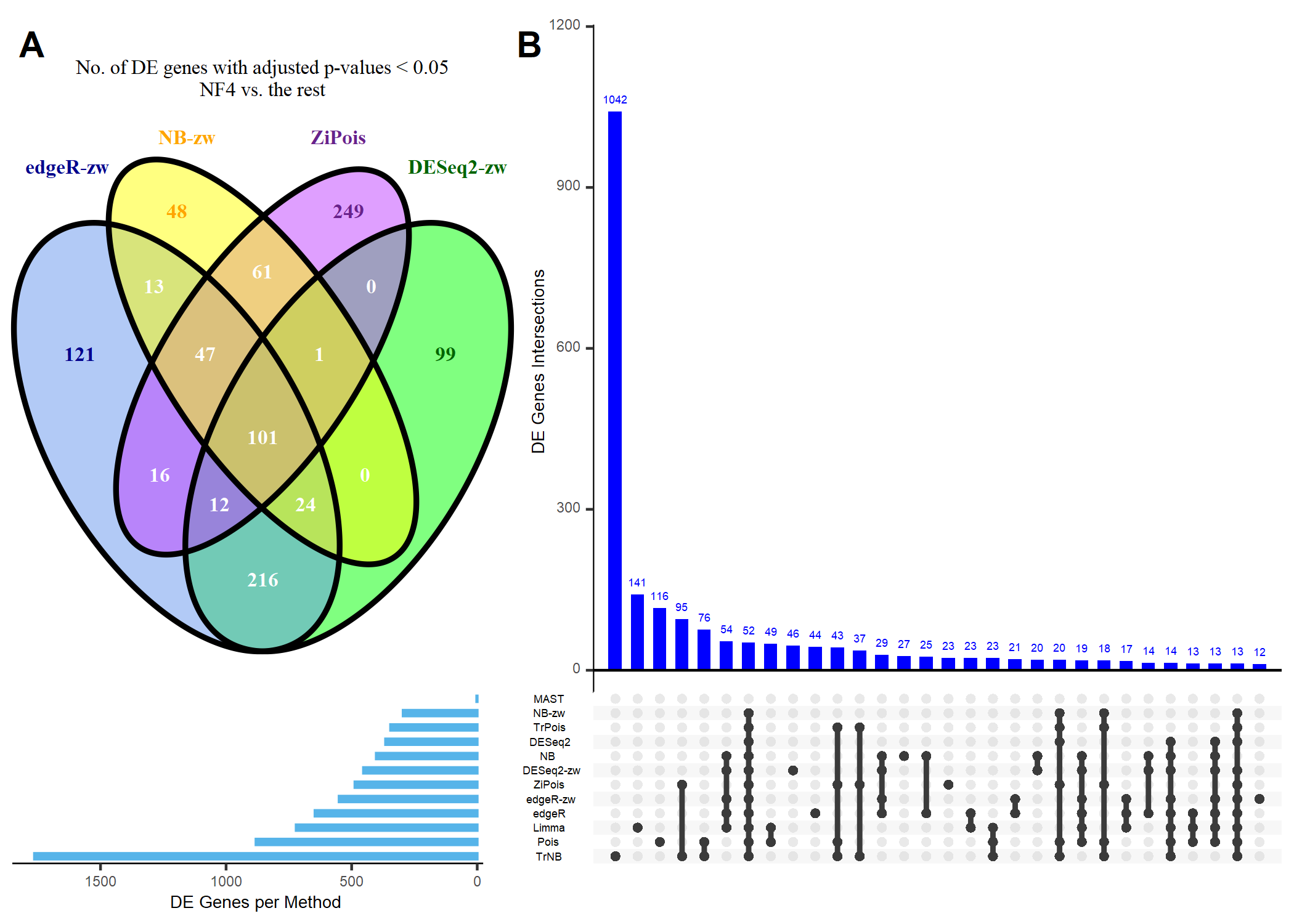

Supplement: Supplementary file 3 [file Presentation_1.zip › Supplementary_Figures/Figure_S8_Usoskin04NF4.tiff]

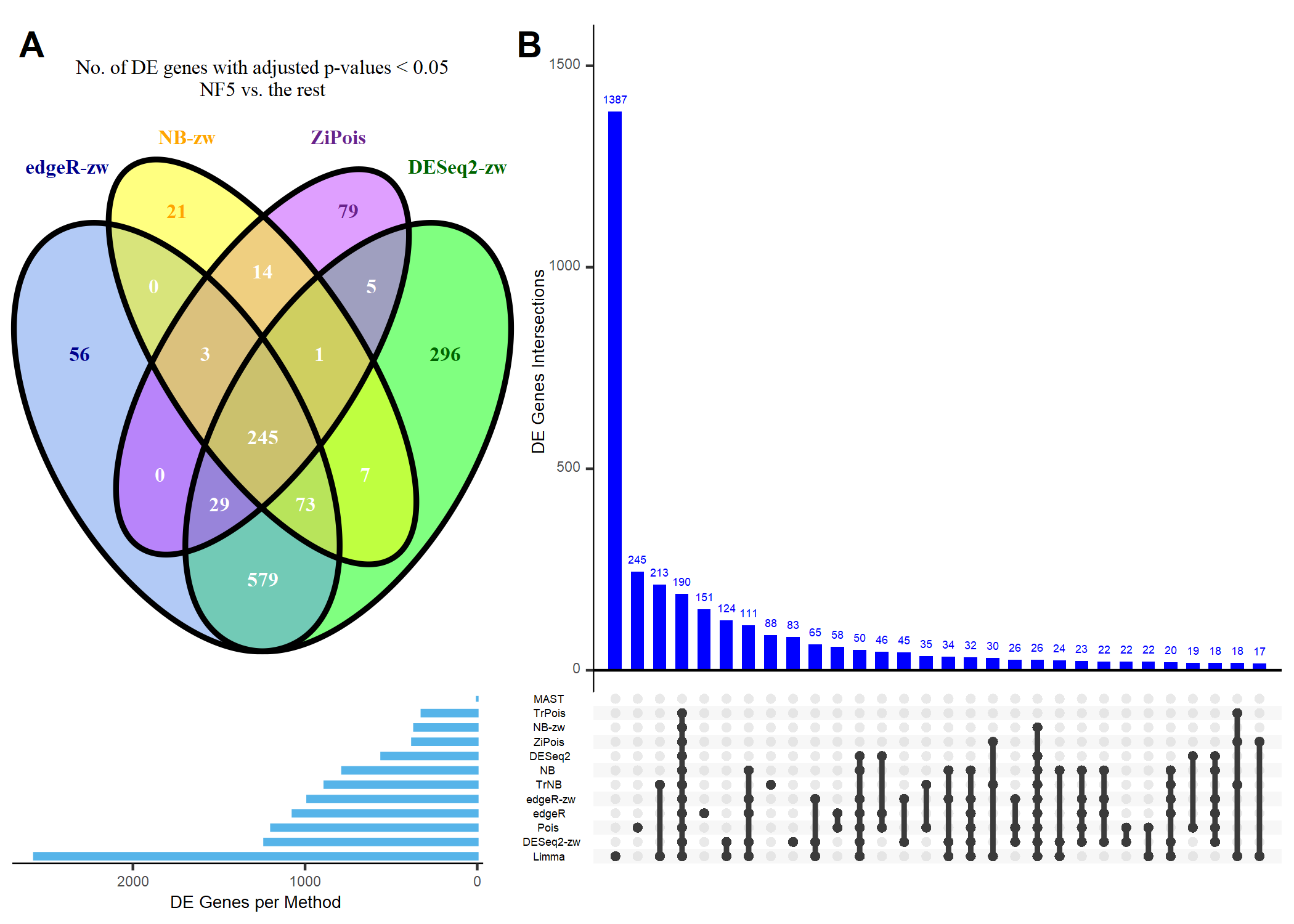

Supplement: Supplementary file 3 [file Presentation_1.zip › Supplementary_Figures/Figure_S9_Usoskin05NF5.tiff]
